# Genomes of *Fasciola hepatica* from the Americas Reveal Colonization with *Neorickettsia* Endobacteria Related to the Agents of Potomac Horse and Human Sennetsu Fevers

**DOI:** 10.1371/journal.pgen.1006537

**Published:** 2017-01-06

**Authors:** Samantha N. McNulty, Jose F. Tort, Gabriel Rinaldi, Kerstin Fischer, Bruce A. Rosa, Pablo Smircich, Santiago Fontenla, Young-Jun Choi, Rahul Tyagi, Kymberlie Hallsworth-Pepin, Victoria H. Mann, Lakshmi Kammili, Patricia S. Latham, Nicolas Dell’Oca, Fernanda Dominguez, Carlos Carmona, Peter U. Fischer, Paul J. Brindley, Makedonka Mitreva

**Affiliations:** 1 McDonnell Genome Institute at Washington University, St. Louis, Missouri, United States of America; 2 Departamento de Genética, Facultad de Medicina, Universidad de la República (UDELAR), Montevideo, Uruguay; 3 Department of Microbiology, Immunology and Tropical Medicine, and Research Center for Neglected Diseases of Poverty, School of Medicine & Health Sciences, George Washington University, Washington, DC, United States of America; 4 Division of Infectious Diseases, Department of Medicine, Washington University School of Medicine, St. Louis, Missouri, United States of America; 5 Department of Pathology, School of Medicine & Health Sciences, George Washington University, Washington, DC, United States of America; 6 Unidad de Biología Parasitaria, Instituto de Biología, Facultad de Ciencias, Instituto de Higiene, Montevideo, Uruguay; Max-Planck-Institut fur Evolutionsbiologie, GERMANY

## Abstract

Food borne trematodes (FBTs) are an assemblage of platyhelminth parasites transmitted through the food chain, four of which are recognized as neglected tropical diseases (NTDs). Fascioliasis stands out among the other NTDs due to its broad and significant impact on both human and animal health, as *Fasciola sp*., are also considered major pathogens of domesticated ruminants. Here we present a reference genome sequence of the common liver fluke, *Fasciola hepatica* isolated from sheep, complementing previously reported isolate from cattle. A total of 14,642 genes were predicted from the 1.14 GB genome of the liver fluke. Comparative genomics indicated that *F*. *hepatica* Oregon and related food-borne trematodes are metabolically less constrained than schistosomes and cestodes, taking advantage of the richer millieux offered by the hepatobiliary organs. Protease families differentially expanded between diverse trematodes may facilitate migration and survival within the heterogeneous environments and niches within the mammalian host. Surprisingly, the sequencing of Oregon and Uruguay *F*. *hepatica* isolates led to the first discovery of an endobacteria in this species. Two contigs from the *F*. *hepatica* Oregon assembly were joined to complete the 859,205 bp genome of a novel *Neorickettsia* endobacterium (*nFh*) closely related to the etiological agents of human Sennetsu and Potomac horse fevers. Immunohistochemical studies targeting a *Neorickettsia* surface protein found *nFh* in specific organs and tissues of the adult trematode including the female reproductive tract, eggs, the Mehlis’ gland, seminal vesicle, and oral suckers, suggesting putative routes for fluke-to-fluke and fluke-to-host transmission. The genomes of *F*. *hepatica* and *nFh* will serve as a resource for further exploration of the biology of *F*. *hepatica*, and specifically its newly discovered trans-kingdom interaction with *nFh* and the impact of both species on disease in ruminants and humans.

## Author Summary

This report presents novel findings revealing (a) the genome sequence of the food-borne trematode *Fasciola hepatica* (the liver fluke) isolated from sheep, which stands out among neglected tropical diseases due to its zoonotic impact on both human and animal health and (b) the first instance (and the genome) of the rickettsial endobacterium of the genus *Neorickettsia* in *F*. *hepatica*. Using stage-specific gene expression data, we identified liver fluke proteins likely involved in host-parasite interactions, and using immunolocalization, we confirmed *Neorickettsia* in organs and tissues of the adult trematode. The presence of the bacteria in fluke reproductive tissues and eggs suggests a possible mechanism for vertical transmission, and the presence of bacteria in the oral sucker used to anchor flukes to the lining of the biliary tract suggests a potential mechanism for horizontal transmission to the mammalian host. This is of interest because related *Neorickettsia* cause severe, even deadly, illness in a variety of species, including humans. This is the first report to localize *Neorickettsia* endobacteria within the tissues of adult *F*. *hepatica*. The discoveries in our manuscript have wide impact for the fields of both the pathophysiology and evolution of *Fasciola* and related FBTs, and the transmission strategies of *Neorickettsia*.

## Introduction

Food borne trematodes (FBTs) are an assemblage of platyhelminth parasites that are transmitted through the food chain [1]. Among the four major groups of FBT infections recognized as neglected tropical diseases (NTDs) by the World Health Organization [2], fascioliasis stands out due to its zoonotic impact on both human and animal health [3]. *Fasciola* species are major pathogens of domesticated ruminants, but they infect numerous other species of mammals, including people [4]. Due to the significant burden to livestock globally, with annual losses exceeding US $3.2 billion [5] and public health with ~50 million infected people [4], these parasites are among the most-extensively studied FBTs.

Like other digenetic trematodes, *Fasciola hepatica* has a complex developmental cycle [1]. The hermaphroditic adult stage resides in the host bile ducts and reproduces sexually, releasing thousands of eggs each day that pass with the bile into the intestines and exit in the fecal stream. Eggs that reach fresh water embryonate over a couple of weeks, hatching a free-swimming miracidium that seeks out and infects a snail of the family Lymnaeidae. Within the snail, the parasite progresses through sporocyst, redia, and daughter redia stages by asexual replication and development, resulting in the release thousands of the cercariae [6]. The free-living, aquatic cercaria encysts as the metacercarial stage on solid substrates, including vegetation at the margins of the watercourse. When infected vegetation (for example, uncooked watercress) are ingested by a suitable host, the metacercaria excysts in the duodenum, transverses the wall of the small intestine, migrates through the peritoneal cavity, and penetrates the Glisson's capsule of the liver [7]. The migration of the juvenile fluke though the liver parenchyma into the biliary ducts damages the liver and provokes reactions associated with the acute phase of the infection. This phase is accompanied by systemic disease including fever, nausea and abdominal pain. Once the adult is established in the bile ducts, anemia, inflammation, fibrosis, cholangitis and biliary stasis may ensue. In this chronic phase adult worms can survive several years in the absence of intervention [8, 9]. Despite its potent and broad action against other human parasitic flatworms the anthelmintic drug praziquantel has no effect on *F*. *hepatica* [10]. Triclabendazole (TCBZ) is the drug of choice since its effective against juveniles and adult liver flukes, but resistance to this benzimidazole has emerged in livestock in different countries [11]. There have been recent reports of human fascioliasis refractive to TCBZ treatment in Peru and Chile [12, 13], highlighting a need for alternative drugs and treatments.

In addition to being important pathogens themselves, some digeneans serve as vectors of bacterial pathogens. *Neorickettsia* (family Anaplasmataceae) belongs to a poorly characterized assemblage of obligate, intracellular α-Proteobacteria associated with serious, even fatal disease in mammals [14]. These bacteria can be horizontally transmitted from the fluke to host tissue invading and multiplying within mammalian cells such as macrophages, monocytes and other cells types, e.g. intestinal epithelium, eventually leading to severe disease. *Neorickettsia* can be detected by PCR in trematodes spanning the major lineages of the Digenea [15, 16], but it has never been reported from a trematode that is itself a prevalent human and livestock pathogen. Furthermore, the fact that *Neorickettsia* is not found among all fluke species (or all members of infected species) suggests that these endobacteria are not essential to fluke survival. Indeed, the exact nature of their relationship is remains unclear.

Here we describe the second reported reference genome of the common liver fluke, *F*. *hepatica* and the first discovery and genome sequences of the *Neorickettsia* endobacteria of *F*. *hepatica*. In contrast to the previously sequenced isolate from cattle from the UK, the presently described strain, taken from a sheep in Oregon, US, was infected with a *Neorickettsia* species closely related to the etiological agents of Potomac horse and Sennetsu fevers. Histological, PCR, and gene sequence analyses revealed its presence in tissues of liver fluke isolates from Oregon, and in one of several liver fluke isolates from Uruguay that were screened. Taken together, these genomes represent a benchmark resource for studies of trematode and *Neorickettsia* biology, pathogenesis and evolution.

## Results and Discussion

### The general features of the nuclear genome of *Fasciola hepatica*

The nuclear genome of *F*. *hepatica* Oregon was sequenced and assembled with a total length of 1.14 Gb, N50 number of 2,036 and N50 length of 161 kb (S1 Table). Completeness was estimated at 90.6% using the CEGMA method [17]. GC content was similar to other Food Borne Trematodes (FBTs) including *Clonorchis sinensis* [18] and *Opisthorchis viverrini* [19], but differed from blood flukes. Intriguingly, the genome of *F*. *hepatica* Oregon had a markedly higher repeat content (55.29%, S1 Table) than other FBTs, including the recently published genome of *F*. *hepatica* United Kingdom (32.0%) isolated from cattle. We detected > 92 Mb corresponding to LTR elements, 268 Mb corresponding to LINEs, and 235 Mb of unclassified repetitive sequences, values all higher than other trematodes. Functional RNAs including rRNA, tRNA and miRNA (S2 Table) were identified, representing 0.002% of the coding genome, most supported by RNAseq data.

Consistent with other FBTs [19, 20], a very small percentage of the genome assembly was predicted to encode proteins (1.08%, considering only exonic regions). A total of 14,642 protein-coding genes were identified using a combination of *de novo* and evidence-based methods. Predicted genes had an average of 3.3 exons and 2.3 introns, average footprint of 3,078 bp, and average coding length of 837 bp (S1 Table). Comparisons of protein-coding genes between *F*. *hepatica* Oregon and *F*. *hepatica* UK (S1 Fig) revealed that most functional elements (e.g. KEGG orthologous groups) were shared, despite the fact that the gene models showed relatively poor overlap. In general, both genome annotations showed a predominance of short genes compared with other trematodes. This could be an indication of incomplete gene models in both assemblies, as the size, complexity, and incompleteness of both hindered gene prediction. Accordingly, long reads from third generation sequencing and additional RNAseq data will be needed to improve gene predictions, as demonstrated for *S*. *mansoni* [21] and *C*. *sinensis* [20].

Comprehensive functional annotation of the deduced proteins of *F*. *hepatica* Oregon is provided in S3 Table, including (a) 3,907 unique InterPro protein domains predicted from 8,609 proteins, associated with 1,147 unique gene ontology (GO) terms, (b) 3,175 proteins associated with 2,685 KEGG orthologous groups, (c) 339 proteins classified as putative proteases, (d) 65 proteins classified as protease inhibitors, and (e) 855 of proteins predicted to be secreted. Majority of the genes (94% of the predicted 13,740/14,642) were supported by RNAseq data from the developmental stages sampled for this study (eggs, metacercariae, and adult flukes; S2 Fig). Of the >6,000 genes expressed in these stages, ~2,500 showed no differential expression, with GO terms related to core cellular functions such as translation, RNA processing, and vesicular transport (S3 Table).

Among the differentially expressed gene sets resulting from the DESeq analysis, stage-specific overexpressed gene sets were identified (e.g., for metacercariae, genes significantly overexpressed in the metacercarial stage relative to the adult and to the egg, but not differentially expressed between adult and egg). Using these criteria, four of the top five most significantly metacercaria-overexpressed genes (2,076 total) were cysteine proteases, including four papain-like family proteases and one C13-family protease (P < 10^−15^ for all comparisons), while the most significantly adult-overexpressed was also a papain-family cysteine protease (P < 10^−38^). Among the other 1,169 adult-overexpressed genes, four of the top 11 were tubulin genes (P < 10^−20^). Fewer genes (259) were egg-overexpressed since adult females contain eggs expressing transcripts, but the most significantly differentially expressed gene in this set (P < 10^−13^) was a glucose-6-phosphate dehydrogenase.

### Comparative analysis reveals phylogenetic conservation and diversification among trematodes

Approximately 88.5% of the 14,642 inferred proteins from the *F*. *hepatica* Oregon isolate found at least one BLAST hit (E < 1e^-05^) to non-*Fasciola* proteins in the non-redundant database (NR), with most matching sequences from other FBTs (particularly the liver flukes *C*. *sinensis* and *O*. *viverrini*). 11.5% of genes are *Fasciola-*specific with respect to NR (S3 Table), 4.9% of which were assigned additional functional annotations (Interpro domains, GO terms or KEGG orthologous groups; compared to 67.7% for genes with non-*Fasciola* NR matches). The putative *Fasciola*-specific genes were enriched for GO terms related to cysteine-type endopeptidase inhibitor activity and neurotransmitter secretion (S4 Table).

**Fig 1:**
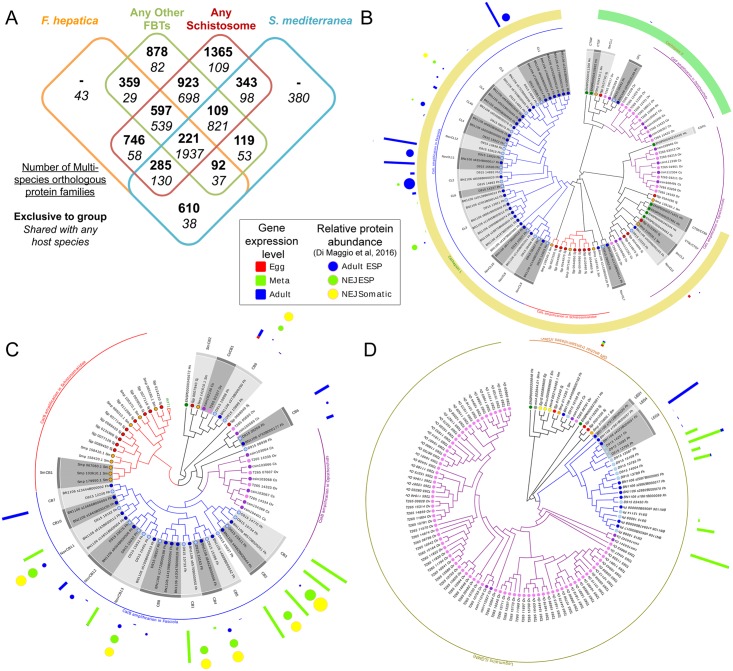
Protein families in trematodes. (A) A Venn diagram demonstrating the phylogenetic distribution of orthologous protein families among the trematode species analyzed (B) Differential amplification of cathepsins L and F in trematodes. A maximum likelihood tree of the genes annotated as members of the C1A protease family from trematodes shows that while a single set of cathepsins F are detected in Schistosomes and *F*. *hepatica* strains, an expansion of cathepsins F is observed in *O*. *viverrini* and *C*. *sinensis* (green arch). Similarly, the cathepsins L-like of the trematodes (yellow arch) show a basal node more related to the mammalian enzymes and several independent amplifications in schistosomes, opistorchiids and *Fasciola*. Most known cathepsins variants are supported by expression data in different stages (red, green and blue bars), and proteomic data (yellow, green and blue dots) from a recent report [34]. Several putative novel variants are indicated, most of them not expressed at the stages analyzed. (C) Cathepsin B subfamily of the C1A protease family. A basal cathepsin B node and differential expansions in schistosomatids (red arch), fasciolids (blue arch) and opisthorchiids (purple arch) is observed. As in cathepsins L novel isoforms are identified, some of them supported by expression data. (D) Legumain is differentially amplified in food borne trematodes. Maximum likelihood tree of the genes annotated as members of the C13 protease family. While a single gene corresponding to the glycosyl-phosphatidyl-inositol anchor transamidase exists in all trematodes, amplification of the legumains was evident in the food borne trematodes *F*. *hepatica*, *C*. *sinensis and O*. *viverrini*, but not in the blood flukes *S*. *mansoni* and *S*. *japonicum*. These amplification events are independent in the different lineages. Species included in the tree are color coded (human, emerald dots, planaria (grey) cestodes (yellow) *S*. *japonicum* (dark red) *S*. *mansoni* (orange) *C*. *sinensis* (purple) *O*. *viverrini* (pink), *F*. *hepatica* Oregon strain (sky blue) *F*. *hepatica* Liverpool strain (navy).

Protein conservation among flatworm parasites and their hosts was analyzed by clustering predicted proteins from 10 genomes into orthologous protein families (OPFs; Fig 1A), and 7,624 *F*. *hepatica* proteins were included in 5,721 unique OPFs with proteins from other flatworms including the free-living planarian *Schmidtea mediterranea*, trematodes (including schistosomes, the liver flukes *C*. *sinensis* and *O*. *viverrini*), and mammalian hosts (human, cow and sheep). Some 2,875 *F*. *hepatica* proteins (1,451 OPFs) were conserved across the 10 species, and these were more likely than other genes to be differentially expressed across developmental stages of *F*. *hepatica* (P = 7 x 10^−5^, binomial distribution test; S3 Table). In contrast, 393 *F*. *hepatica* genes (359 OPFs) were conserved between *F*. *hepatica* and at least one other FBT (*C*. *sinensis* and/or *O*. *viverrini*); these were significantly enriched for GO terms related to microtubule-based processes, cysteine endopeptidase activity and pH regulation (S4 Table). An additional 29 genes (29 OPFs) were conserved with at least one FBT and at least one host species; these were significantly enriched for the GO terms related to phagocytosis and L-ascorbic acid binding.

OPF analysis also enabled the identification of gene sets specific to schistosomes; 1,365 OPFs conserved among at least two of the three species of *Schistosoma* were analyzed. Like the FBTs, these were significantly enriched for GO terms related to microtubule activity and cysteine endopeptidases (see below). This suggests that certain functions are conserved across the platyhelminth clades despite clear divergence at the sequences level, a possible indication of rapid evolution [22].

### Expanded families of secreted/excreted proteases suggest a role in host-parasite interaction

Excreted and secreted proteins (ESPs) play a crucial role in parasitism. We identified 855 (5.8%) proteins with computationally predicted signal peptides but no transmembrane domains (S3 Table), indicating they may be secreted/ excreted. These proteins were significantly enriched for GO terms related to proteolysis (S4 Table). Again, the most significantly enriched molecular process GO term was “cysteine-type endopeptidase activity” (GO:0004197). Secreted cysteine proteases have a well-defined role in the biology of *F*. *hepatica* and liver fluke disease [23]. Cathepsin L’s are predominant in adult ESPs, where they participate in feeding, immune evasion and immune modulation. Distinct suites of cathepsin L’s and cathepsin B’s are abundant in the juvenile fluke, participating in excystment, migration through gut wall and liver capsule, and immune evasion [24, 25]. Although it was known that liver fluke cathepsins constitute a multigene family [26], the complexity and diversity within the family is now apparent. In addition to the six known cathepsin L’s, other isoforms were detected consistently in both the Oregon and the UK isolates, raising the total count to 14; most of these overexpressed in the adult stage (Fig 1B). Independent amplifications of cathepsin L’s occurred in schistosomes and the opisthorchiids, but the resulting gene copy number is less than in *F*. *hepatica*. In contrast, Cathepsin F’s showed a divergent pattern in these lineages, with single enzymes in *Fasciola* and schistosomes, and an amplified family in the carcinogenic fish-borne liver flukes (Fig 1B). A similar pattern of independent amplifications among trematodes was observed for cathepsin B’s (Fig 1C), again with a distinct expansion in *F*. *hepatica*. In contrast to cathepsin Ls, cathepsin Bs were overexpressed in metacercariae (MC), confirming biochemical, genetic and proteomic evidence of differential expression along the life cycle [27]. Interestingly, within both the cathepsins L and B, a clade comprising a single enzyme from each trematode species and vertebrates was identified, which might be basal to all the lineage-specific expansions. *F*. *hepatica* enzymes of this clade have not been described yet, and, notably, they are expressed in eggs (Fig 1B and 1C).

The remarkable amplification and diversity of secreted cysteine proteases in trematode lineages suggests key roles during parasite adaptation. Diverse trematodes express different (and amplified) subfamilies of cathepsins, reflecting their host parasite relationships, including host niche, organ sites, and transmission strategies. For example, cathepsins B and an L3 (CL3) participate in transit of the juvenile liver fluke through the gut wall with collagenolysis [28, 29], whereas juvenile of the fish-borne liver flukes ascend into the biliary tree through the ampulla of Vater [30]. In turn, cathepsins F and the aspartic protease cathepsin D are characteristically overrepresented in these carcinogenic liver flukes [19, 31]. The blood flukes, on the other hand, invade the skin of their hosts, with conserved serine proteases essential to this process, with the cysteine protease cathepsin B providing critical activities in some species [32].

Asparaginyl endopeptidases, Class C13 (also known as legumain) [33] were expanded in *F*. *hepatica* with ≥10 members, and were also differentially expanded among the trematodes; 3 copies in *S*. *mansoni*, 5 in *S*. *japonicum*, 4 in *C*. *sinensis* and ~ 100 in *O*. *viverrini*. (Fig 1D). These proteases might participate in the activation of cathepsins and the digestion of infected host tissues, liberating essential amino acids.

A recent proteomic study of ESPs from juvenile and adult *F*. *hepatica* provide further support for the differential expression of these gene families [34], confirming our transcriptomic data (S3 Table). For example, within the cathepsin B family, members of 10 out of 13 clusters are detected by LC-MS/MS in ESPs; while 3 isoforms are exclusively expressed by adults, 2 are characteristic of juveniles (also detected by RNAseq in metacercariae), and 5 are expressed in both stages but clearly predominant in juveniles (Fig 1C). Similarly, a predominance of expression of cathepsin Ls variants in adults is observed at proteomic level consistent with our transcriptomic data (Fig 1B). Within these some of the novel clades here described were detected as being expressed.

### FBTs are metabolically less constrained than blood flukes and cestodes

**Fig 2:**
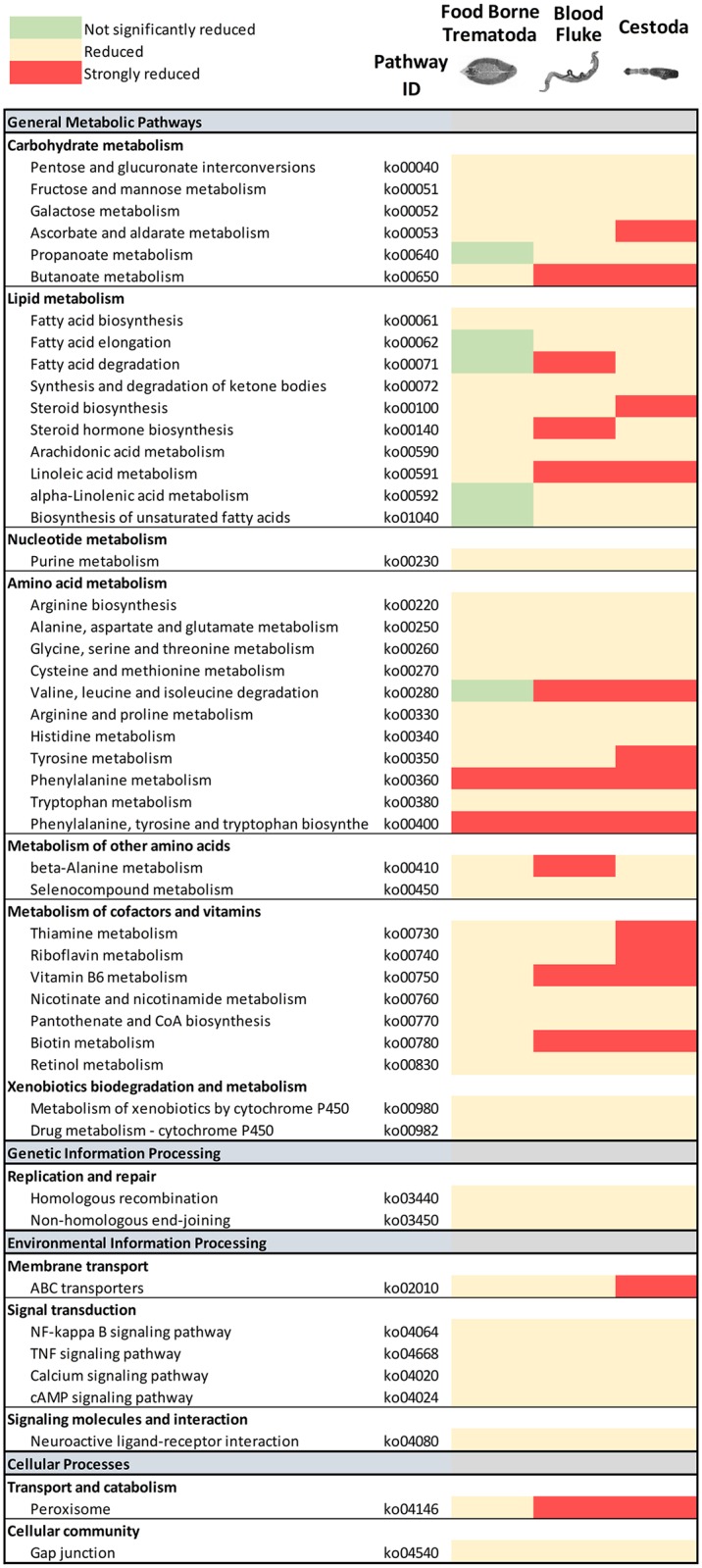
Metabolic pathway reduction in parasitic flatworms. The global number of proteins assigned to different metabolic pathway was compared between different parasitic flatworms, the free-living planaria and cattle predicted proteomes. Those pathways showing a significant reduction in the parasitic species in relation to those present in planaria are indicated (strongly reduced: less than 30% conservation; reduced: conservation between 30 and 80%; not significantly reduced: more than 80% conservation). While in general several pathways are reduced in all parasitic species, some pathways are differential between food borne trematodes (*F*. *hepatica*, *O*. *viverrini*, *C*. *sinensis*), blood flukes (*S*. *mansoni* and *S*. *japonicum*) and cestodes (*E*. *multilocularis*, *H*. *microstoma* and *T*. *solium*). Most notably some lipid metabolism and amino acid pathways (i.e. aliphatic amino acid degradation) are not reduced in FBT (green) while they are reduced in the other groups. In general, FBT seem to be less constrained than blood flukes, with cestodes being the most restricted metabolically.

Metabolic pathways predicted in the *F*. *hepatica* Oregon strain were compared to those of other sequenced flatworms. All parasitic flatworms showed a significant reduction in metabolic capabilities compared to free living platyhelminth species, including planaria (Fig 2). As shown previously [35], parasitic flatworms depend on the hosts for provision of fatty acids. Unlike the blood flukes, however, *F*. *hepatica* and the other liver flukes possess enzymatic pathways for fatty acid elongation by reversal of beta-oxidation (S3A Fig) and fatty acid catabolism (S3B Fig), allowing them to take advantage of the fatty acid rich environment of bile.

Additional differences between blood and liver flukes were evident in amino acid metabolism. Inabilities to synthesize several amino acids were generally observed in neodermatan flatworms, including *Fasciola*. However, the liver flukes operate a complete catabolic pathway of aliphatic amino acids, and enzymes of these pathways (e.g. branched chain amino acid aminotransferase [BCAT, EC. 2.6.1.42]) are missing in schistosomes (S3C Fig). Aliphatic amino acids are more abundant in the bile than in blood, which may have been exploited since it facilitates access to protein synthesis precursors and alternative energy sources. While expanded families of secreted proteases are involved in protein digestion, conserved oligopeptide transporters that mediate the uptake of di- and tri-peptides in metazoans were not identified [36], suggesting the lack of these specific transporters in trematodes at large. Consequently, protein digestion up to individual amino acids may occur extracellularly, explaining the rare presence of usually cytoplasmic enzymes as leucine aminopeptidase in the secreted products and vesicles released by the parasite [37].

**Fig 3:**
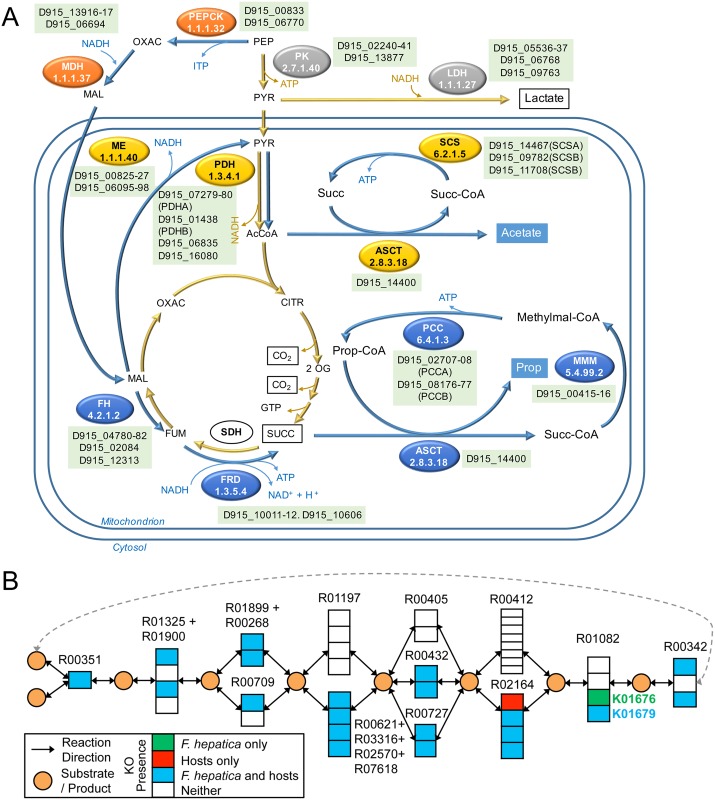
Metabolic pathways in *F*. *hepatica*. (A) Energy metabolism in anaerobic mitochondria of *F*. *hepatica* by malate dismutase. While the classical anaerobic fermentation to lactate is present (enzymes indicated in gray) when oxygen tension is low, the malate dismutation pathway is preferred (blue arrows). Phosphenol pyruvate reduction to malate occurs in the cytoplasm (orange). Within the mitochondria, part of the malate is oxidized to acetate (yellow) while other fraction reduced to succinate and further transformed to propionate (blue). Genes predicted for key enzymes involved in anaerobic respiration are indicated. Abbreviations: PEP, phosphenolpyruvate; OXAC, oxaloacetate; MAL, malate; FUM, fumarate; SUCC, succinate; PYR, pyruvate; AcCoA, acetyl-CoA; CITR, citrate. Enzymes indicated are: PK, pyruvate kinase; LDH, lactate dehydrogenase, PEPCK, phosphenolpyruvate carboxykinase (ATP dependent); MDH, malate dehydrogenase; ME, malic enzyme; PDH, pyruvate dehydrogenase; ASCT, acetate:succinate CoA-transferase; SCS, succinyl-CoA synthetase; FH, fumarate hydratase; FRD, fumarate reductase; SDH, succinate dehydrogenase, MMM methylmalonyl-CoA mutase; PCC, propionyl-CoA carboxylase. (B) Parasite specific enzyme usage in TCA cycle. The KEGG module for TCA cycle (M00009) is shown with groups of orthologous enzymes indicated using KEGG orthology (KO) IDs. An interesting example of alternate enzyme usage is shown for fumarate hydratase (reaction R01082), catalyzed by a class II enzyme (EC 4.2.1.2B; K01679) in both the host and parasite. However, *F*. *hepatica* also has a Platyhelminthes-specific class I fumarate hydratase (EC 4.2.1.2A; K01676), not annotated in any of the host proteomes. Such knowledge can be leveraged to design worm specific therapies with potentially low (or no) impact on the host health.

Further metabolic differences may have evolved in liver flukes compared to blood flukes in relation to an environment characterized by low oxygen tension. Flukes switch from aerobic to anaerobic metabolism in the low oxygen environment of the bile duct, but instead of fermenting carbohydrates to lactate, the parasite exploits the more energy-efficient malate dismutation pathway [38], where phosphoenolpyruvate from glycolysis is converted to oxaloacetate via the phosphoenolpyruvate kinase (PEPCK), and further reduced to malate. After entering the mitochondria, some malate is oxidized to acetate, and some is reduced to succinate and transformed to propionate, in a series of reactions that reverts the Krebs cycle (Fig 3A), providing a source of electrons for the respiratory chain finally yielding five ATP molecules per glucose molecule. While the whole pathway was precisely described biochemically, we now for the first time identify the cognate enzymes (Fig 3A).

To provide further insight into the metabolism of *F*. *hepatica*, we compared the metabolic pathway modules present and complete in *F*. *hepatica*, the carcinogenic liver flukes *C*. *sinensis* and *O*. *viverrini*, the blood fluke *S*. *mansoni* and mammalian hosts of *F*. *hepatica* (sheep, cow and human) were incorporated in the analysis (S4A Fig). Twenty-five KEGG pathway modules were complete in our assembly (i.e., contain the complement of KO’s necessary to convert the initial substrate to the final product based on strict completion [39]). These values are similar in other trematodes, but far lower than in mammals. Use of a lenient completion criterion of ≤2 missing steps in a module extended these values, with similar trends (S4B Fig). The analysis also identified modules that differ between the liver flukes and *S*. *mansoni*. Module M00020 (serine biosynthesis) also showed differences consistent with those observed in amino acid metabolism. Further differences in inositol phosphate metabolism were detected with two steps missing in *S*. *mansoni* (R03427 and R04372, INPP1 and INPP4 phosphatases). Module M00087 (beta-oxidation) occurs in liver flukes but is absent from schistosomes. Notably, this module revealed differences with the host, since in step 2 (R04738), *F*. *hepatica* shares two KOs with mammals, but there is an additional, putative platyhelminth-specific ortholog (K01692, EC 4.2.1.17) corresponding to enoyl coA-hydratase, which warrants investigation in flatworms. Differences between liver fluke and mammal were evident in module M00009m, corresponding to the tricarboxylic acid cycle (Fig 3B). The fumarate forming reaction (R01082) is dependent on fumarate hydratase class II enzyme (EC 4.2.1.2B, K01679) by the hosts, while a second fumarate hydratase, class I enzyme for this step (EC 4.2.1.2A, K01676), was detected in trematodes, an enzyme that might participate in the reverse step of the malate dismutation pathway (above).

### A complete *Neorickettsia* genome identified in *F*. *hepatica* Oregon and Uruguay

**Fig 4:**
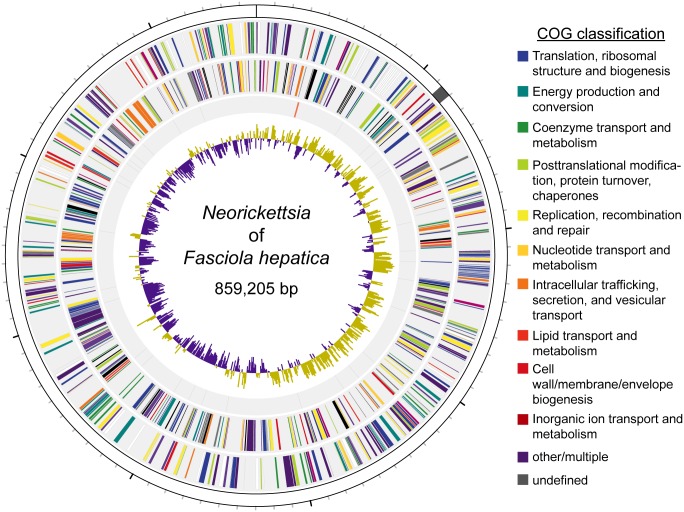
The genome of the *Neorickettsia* endobacterium of *Fasciola hepatica*. The first track (from outside to inside) represents the 859,205bp genome of the *Neorickettsia* endobacterium of *Fasciola hepatica* (100 kb major ticks, 10 kb minor ticks). The genome shows nearly complete synteny with *Neorickettsia risticii* and *Neorickettsia sennetsu* with the exception of an inversion (shaded in grey). The second and third tracks represent the 744 inferred protein coding genes on the plus and minus strands, respectively. Genes are coded based on their NCBI Clusters of Orthologous Groups of proteins database classification. The fourth track represents RNA coding genes, including 3 ribosomal (red), 33 transfer (black), and 1 short, noncoding (blue). The fifth track depicts the G-C skew [(G-C)/(G+C)] calculated over 500bp windows.

**Fig 5:**
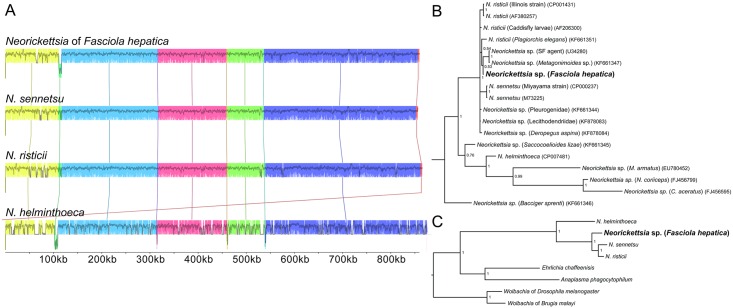
Phylogenetic affinities of the *Neorickettsia* symbiont of *Fasciola hepatica*. (A) The genomes of the four fully sequenced *Neorickettsia* species were aligned. Syntenic blocks are colored. The genome of the *Neorickettsia* of *Fasciola hepatica* (PRJNA295290) shares almost complete synteny with the genomes of *Neorickettsia risticii* (PRJNA19099) and *Neorickettsia sennetsu* (PRJNA357), with the exception of a small inversion that is also present in *Neorickettsia helminthoeca* (PRJNA187358). (B) Bayesian inference phylogenetic analysis based on available 16S ribosomal RNA sequences of *Neorickettsia* species, retrieved from [15]. While the resolution of the sub-clade consisting of *Neorickettsia risticii* and *Neorickettsia* *sennetsu* is sub-optimal, the tree indicates that the *Neorickettsia* of *Fasciola hepatica* (*nFh*) may be most closely related to a strain that occurs in *Metagonimoides* species and an agent of Sennetsu fever. (C) A Bayesian inference phylogenetic tree based on the protein sequences of 473 single-copy, orthologous protein families conserved in the represented species clearly indicates that *nFh* is more closely related to *N*. *risticii* and *N*. *sennetsu* than *N*. *helminthoeca* or other species of the family Anaplasmataceae. NCBI GenBank accession numbers are indicated. Trematode hosts or other defining features are indicated for uncharacterized species.

The most striking feature of the *F*. *hepatica* Oregon isolate was an apparent infection with *Neorickettsia* endobacteria (*nFh*). Alpha-protobacterial sequences were first identified among “contaminating” sequences in the *F*. *hepatica* genome and the presence of *Neorickettsia* was confirmed and validated by both PCR and 16S rRNA sequencing (S5 Fig). The genome of *nFh* was assembled from 241,957 2x100bp read pairs that were identified during the sequencing of *F*. *hepatica* Oregon. A single 859,205 bp scaffold with average 56.3x sequence coverage was constructed from two contigs joined by 189 bp of inferred gaps (Fig 4). This novel *Neorickettsia* genome was similar in size and GC content to those of previously sequenced *Neorickettsia* species [40, 41]. Full genome alignments indicated that, with the exception of a small inversion, it shared nearly complete synteny with the genomes of *Neorickettsia risticii* and *N*. *sennetsu* (Fig 5A). Synteny among *Neorickettsia* species may reflect the lethality of large genome rearrangements due to a reduced set of DNA repair genes, but this may have increased the genetic variation in a stable intra-trematode environment by accumulation of mutations in non-essential genes [41].

Table 1 outlines the inferred features of *nFh*. Similar to related species, *nFh* encodes 33 tRNA genes and one copy each of 5S, 16S, and 23S rRNA genes. A total of 744 protein-coding genes were predicted, slightly fewer than *N*. *risticii* and *N*. *sennetsu* (Table 1). Gene conservation analysis among representative bacterial species of the Anaplasmataceae identified three orthologous protein families (OPFs) that were conserved in all analyzed species except *nFh* (S5 Table). Closer inspection revealed that these genes may be present and intact, although absent from the gene calls. Two additional OPFs were conserved in all sequenced *Neorickettsia* except *nFh* (S5 Table), but in both of these cases the corresponding sequences were identified but stop codons appeared to disrupt them.

**Table 1:** The sequenced genomes of *Neorickettsia* species.

	*Neorickettsia* of *F*. *hepatica*	*Neorickettsia sennetsu*	*Neorickettsia risticii*	*Neorickettsia helminthoeca*
Trematode host	*F*. *hepatica*	Unknown	*Acanthatrium orgonense*	*Nanophyetus salmincola*
Vertebrate host	Unknown	Human	Horse, bat	Canids
Disease	Unknown	Sennetsu fever	Potomac horse fever	Salmon poisoning of dogs
RefSeq Accession	NZ_AGCN 00000000.1	NC_007798.1	NC_013009.1	NZ_CP007481.1
Assembly size	859,205 bp	859,006 bp	879,977 bp	884,232 bp
Length of inferred gaps	189 bp	---	---	---
GC content	41.4%	41.1%	41.3%	41.7%
rRNA	3	3	3	3
tRNA	33	33	33	33
ncRNA	1	3	1	1
Pseudo genes	8	2	11	17
Protein coding genes	744	753	759	772
Average CDS length	968 bp	966 bp	962	970
Minimum CDS length	4,761 bp	5,766 bp	4,761 bp	4,776 bp
Maximum CDS length	156 bp	156 bp	147 bp	135 bp
% coding	83.8%	84.7%	82.9%	84.6%

S5 Table presents a functional annotation of the 744 predicted proteins of *nFh*, including (a) 1,453 unique InterPro protein domains predicted from 620 proteins and associated with 596 unique gene ontology (GO) terms, (b) 720 proteins associated with 509 KEGG orthologous groups, further binned into 120 enzymatic pathways and 101 pathway modules, (c) 25 proteins classified as putative proteases, (d) two protease inhibitors, and (e) 25 proteins with secretion signals (which were enriched for biological process GO terms related to proteolysis and protein transport; S4 Table). Protein transporters such as porins, identified in previous proteomic studies might transport nutrients from the host cytoplasm [42]. Whether *Neorickettsia* enzymes interact with those of the fluke is a fascinating but unresolved question. Revealingly, however, *N*. *risticii* synthesizes nucleotides, vitamins, and cofactors that the fluke cannot, raising the possibility that they may be harvested by the trematode for their mutual advantage [41].

**Fig 6:**
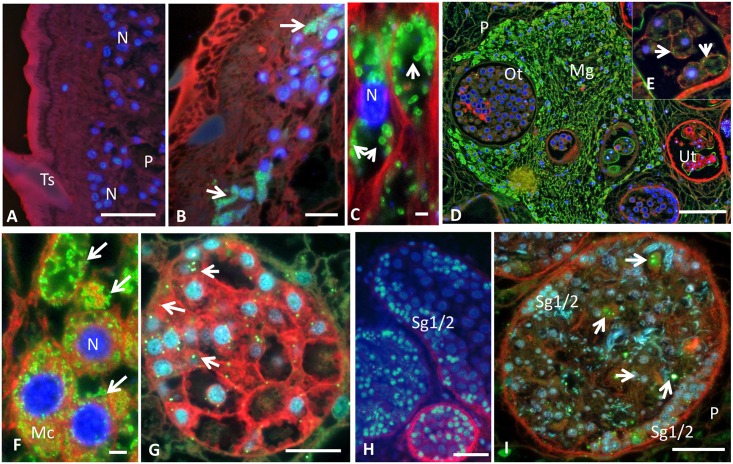
Immunofluorescence detection of *Neorickettsia* in adult *Fasciola hepatica* using polyclonal anti-serum raised against a recombinant surface protein of *Neorickettsia* of *P*. *elegans* (PeNsp-3, green labeling). DAPI (blue) and wheat hemagglutinin (red) were used to detect double stranded DNA and plasma membranes, respectively. (A) No green labeling was seen in the tegument of *F*. *hepatica* from Uruguay that were known to be devoid of *Neorickettsia*. (B) Clusters of *Neorickettsia* (arrows) in the tegument close to tegumental nuclei in *F*. *hepatica* from Oregon. (C) Numerous ‘donut’-shaped endobacteria (arrows) in the parenchyma in *F*. *hepatica* from Oregon. (D) Labeling of large numbers of *Neorickettsia* in the Mehlis’ gland and labeling of single endobacterium in the ootype or intrauterine eggs of *F*. *hepatica* from Oregon. (E) Magnification of a region proximal to (D) showing granular staining of single endobacterium in intrauterine eggs. (F) Clusters of *Neorickettsia* rods (arrows) in the cytoplasm of Mehlis’ cells of *F*. *hepatica* Oregon. (G) Individual *Neorickettsia* endobacteria (arrows) in a vitelline follicle with different stages of vitelline cells of *F*. *hepatica* Oregon. (H) No green staining indicative of *Neorickettsia* were found in the testis of *F*. *hepatica* from Uruguay tested negative for *Neorickettsia* by PCR. (I) *Neorickettsia* endobacteria (arrows) in the testis of *F*. *hepatica* Oregon with spermatogonia in the periphery and developing spermatozoa in the center. N, nucleus; Ts, tegument spine; P, parenchyma; Mg, Mehlis’ gland; Ot, ootype; Ut, uterus; Mc, Mehlis’ cell; Sg1/2, primary and secondary spermatogonia; Bar corresponds in A-B, D-I to 100 μm and in C to 1 μm.

While sequencing reads from the previously reported UK strains (SRA Project ID: ERP006249) did not map to our *nFh* genome (Fig 6), suggesting that no *Neorickettsia* DNA was present in the samples, one of our Uruguay isolate (out of five that were screened) tested positive for the presence of *Neorickettsi*a by 16S rDNA PCR (S5 Fig). Whole genome sequencing of this sample recovered the genome of *nFh* (99.9% breadth of genome coverage; S6 Table), allowing a comparative analysis of sequence variation in both the *Neorickettsia* and the fluke genomes. In total, 15 single nucleotide variants were identified between the two *nFh* genomes, 11 of which occurred within the coding regions (7 non-synonymous and 4 synonymous variants; S7 Table). Notably, 2-C-methyl-D-erythritol 4-phosphate cytidylyltransferase (EC 2.7.7.60; AS219_00645), a key enzyme in the MEP pathway of isoprenoid biosynthesis [43], was found among the genes that harbored non-synonymous SNPs. It has been hypothesized that the *Wolbachia* endosymbionts of *Brugia malayi* and *Dirofilaria immitis* rely on their helminth host for the completion of the MEP pathway [44, 45]. Additionally, in many pathogenic and opportunistic bacteria, the MEP pathway intermediate (HMB-PP) has the capacity to modulate vertebrate host immune response [46], suggesting an interesting possibility of the involvement of the isoprenoid biosynthesis pathway in the host-parasite-endosymbiont interactions. Overall, the genetic distance (1-ibs, identity by state) between the Oregon (US) and Uruguay (UY) *nFh* genomes (1.75 × 10^−5^) was three orders of magnitude lower than that estimated between the respective nuclear genomes of *F*. *hepatica* (1.08 × 10^−2^; S8 Table). The observed level of genetic divergence between the US and UY isolates indicated that these flukes are not substantially more closely related to each other than either is to the five published UK isolates (S8 Table) although their *Neorickettsia* endosymbionts are genetically close to each other.

### Phylogenetic affinities of *Neorickettsia* endobacteria of *F*. *hepatica*

A phylogenetic analysis was undertaken using the 16S rRNA sequences from *nFh* and 16 other species and isolates; the findings were similar to those previous reports [15]. Based on the 16S rRNA locus, *nFh* is closely related to the agent of Sennetsu fever (a strain of *N*. *sennetsu*) and a *Neorickettsia* isolate isolated from species of *Metagonimoides* (Heterophyidae) (Fig 5B). A complementary phylogenetic analysis was undertaken with conserved, single-copy homologues from sequenced species of *Neorickettsia* and representatives of the Anaplasmataceae (Fig 5C); in regard to this collection of 473 gene families, *nFh* appeared to be closer to *N*. *risticii* and *N*. *sennetsu* than to *N*. *helminthoeca*, the agent of salmon poisoning in dogs, consistent with synteny-based observations.

Approximately 97% of the predicted proteins of *nFh* showed BLASTP matches in NR (e-value ≤ 1e-05, S5 Table). The top hits were to *N*. *risticii* or other *Neorickettsia* species. Indeed, most *nFh* genes (721 of 744) belonged to 719 OPFs shared with other *Neorickettsia* species (S5 Table). Of the 22 genes that were excluded from OPFs, 17 failed to find a match in NR and most lacked other functional annotations; the other five matched to hypothetical proteins from *N*. *risticii* and *N*. *sennetsu*. A single OPF was identified with members from *nFh*, *Wolbachia* species, *Anaplasma phagocytophilum* and *Ehrlichia chaffeensis*. The *nFh* gene assigned to this OPF was annotated as a replicative DNA helicase. Further assessment will be needed to validate these genes and explore their roles.

A set of 625 OPFs contained members common to all four sequenced *Neorickettsia* genomes; 83 of these OPFs were specific to *Neorickettsia*. The *nFh* proteins included in the *Neorickettsia*-specific OPFs were enriched for cellular component GO terms related to the outer membrane and biological process GO terms related to transport (S4 Table). The association with the cell surface suggested a role in endobacterial-digenean host interactions.

### *Neorickettsia*-like endobacteria localized within tissues of *F*. *hepatica* Oregon

Although reports of *Neorickettsia-*infected trematodes have emerged, they relied on detection by PCR; localization within the trematode was poorly established. Gibson *et al*. [47] employed Ig from the serum of a horse infected with *N*. *risticii* to detect *Neorickettsia* in eggs from the bat-infecting trematode *Acanthatrium oregonense* to support the hypothesis of vertical transmission. The same serum was used to localize *Neorickettsia* in discrete developmental stages of *Plagiorchis elegans*, a trematode of rodents and birds [14]. We attempted to use the horse serum to localize *nFh* in adult *F*. *hepatica* Oregon, but background staining interfered with interpretation of the signals. However, polyclonal antibodies raised against recombinant surface protein-3 of *P*. *elegans Neorickettsia* (*PeN*sp-3) provided useful to support localization studies (Fig 6). The *PeN*sp-3 protein (Genbank KX082665) and of *nFh* (AS219_03540; S5 Table) share 98% identity. Minimal background signal occurred with the *PeN*sp-3 antisera, and *nFh* were sensitively detected as a ‘donut’-shaped structure surrounding the blue DAPI-stained nucleus, consistent with the staining pattern expected for surface proteins (Fig 6C) [48]. Whereas staining of *Neorickettsia* surface protein was not observed in *Neorickettsia*-negative (as confirmed by PCR; Fig 6A and 6H) *F*. *hepatica* from Uruguay endobacteria were detected in six of six individual adult *F*. *hepatica* worms from Oregon.

**Fig 7:**
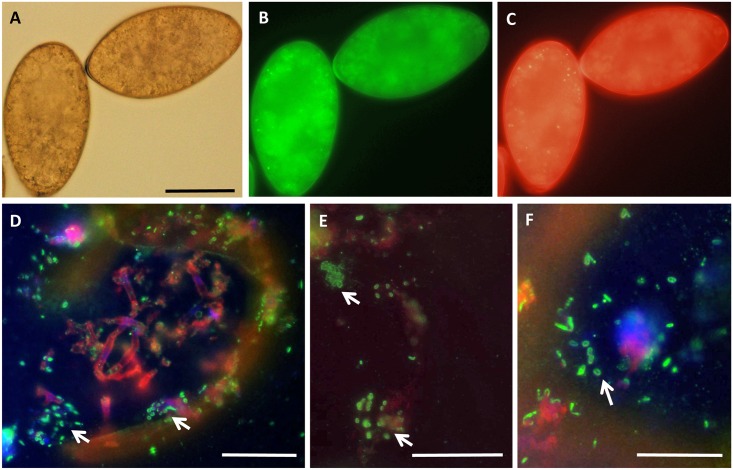
Immunofluorescence detection of *Neorickettsia* in eggs of *F*. *hepatica* from Oregon using polyclonal anti-serum raised against a recombinant surface protein of Neorickettsia of *P*. *elegans* (PeNsp-3, green labeling, D-F). (A) Unstained eggs recovered from the liver by regular light microcopy. (B) and (C) Unstained eggs recovered from the liver by immunofluorescence microscopy using different filters demonstrating auto-fluorescence. (D-F) Cross-sections of eggs showing various amounts of *Neorickettsia* (arrows). Bar corresponds to 50 μm.

Because of the size of *F*. *hepatica* (~2 cm; F) and because *Neorickettsia* may be transmitted vertically, analysis focused on intra-uterine eggs and reproductive tissues. Endobacteria were frequently detected in varying numbers in the ovary, ootype, Mehlis’ gland, vitelline glands and in intrauterine eggs (Fig 6D–6G) as well as mature eggs isolated from liver tissue (Fig 7). The presence of *nFh* in female reproductive tissue is highly suggestive of vertical transmission. Furthermore, we analyzed by PCR adult flukes obtained after an experimental infection with Oregon metacercariae, detecting a few individual worms positive for the presence of nFh (S7 Fig). More interestingly, eggs collected from this assay were both PCR positive and presented the characteristic images of *Neorickettsia* supports the notion of vertical transmission.

Surprisingly, we also observed *nFh* in the testis and other parts of the male reproductive organs (Fig 6I and S8 Fig). Although *F*. *hepatica* is a hermaphrodite, cross-fertilization is assumed to be the usual reproductive strategy [49]. The presence of *Neorickettsia* in spermatozoa and seminal fluid could provide an alternative route for fluke-to-fluke transmission, as it was described for tick-borne pathogens [50], though further studies will be needed to explore this possibility.

The somatic tissues of *F*. *hepatica* Oregon were mostly *Neorickettsia*-free. Clusters of *nFh* were occasionally seen in the tegument adjacent to some syncytial nuclei (Fig 6B) and in intestinal tissue, particularly near the oral suckers. Liver flukes use the oral suckers to penetrate the host tissues and anchor themselves to the bile ducts, thus providing a potential mechanism for fluke-to-host transmission of *nFh*. Several infectious diseases described in the medical and veterinary literature are attributable to *Neorickettsia* carried by digenean parasites; among the more relevant are the ‘Salmon Poisoning Disease’ (SPD) of dogs and the Elokomin fluke fever (EFF) of fish-eating mammals in the west coast of North America, Sennetsu fever described in humans mainly in Japan and southeast Asia, and Potomac Horse Fever (PHF) in the east coast of North America [15, 16]. Notably, PHF, also known as ‘churrido equino’, has been described in horses in the Lake Merin region of Uruguay and Brazil (reviewed in [51]). Several species of *Neorickettsia* based on pathology, serology, antigen profile and/or genomic sequence, are considered the causative agents for these diseases; in particular, *Neorickettsia* (*Ehrlichia*) *sennetsu* causes acute, debilitating, mononucleosis-like disease [52], and has been implicated as a significant cause of human fevers of unknown etiology in southeastern Asia [53, 54].

Whereas the disease potential of *Neorickettsia* found in *F*. *hepatica* remains to be established, the *F*. *hepatica*-*nFh* association should be explored as a cryptic rickettsial pathogen of humans and ruminants in regions endemic for fasciolosis [55]. Additionally, more thorough studies of both the vertical transmission of *nFh* among the developmental stages of the liver fluke, and the potential horizontal transmission to the mammalian host might shine a light on the mechanisms behind the pathology induced by *Neorickettsia* endosymbionts of digenean parasites.

## Methods

### Liver flukes

Two isolates of *Fasciola hepatica* were analyzed: adult worms collected from livers of naturally infected sheep from a commercial slaughterhouse in Oregon (provided by Baldwin Aquatics Inc., Monmouth, Oregon), i.e. Oregon isolate; and worms isolated from livers of naturally infected sheep obtained from a commercial slaughterhouse in Montevideo, Uruguay, i.e. Uruguay isolate. For transcriptomic analysis, total RNAs were obtained from the egg, metacercarial and adult developmental stages (in duplicate). Eggs were collected from gall bladder of naturally infected sheep. Metacercariae were purchased from Baldwin Aquatics Inc. (Monmouth, Oregon). Tissue sections for the histological analysis were prepared from adult worms of the Oregon and Uruguay isolates.

### DNA & RNA isolation, sequencing, assembly, annotation of the genome of *F*. *hepatica* Oregon isolate and transcriptome analysis

Fresh or ethanol-preserved adult worms were fragmented using a scalpel blade, and genomic DNA (gDNA) was extracted and purified using the kit E.Z.N.A. SQ Tissue DNA Kit (Omega Bio-tek), and the yield and purified assessed by Bio-Analyzer. Whole genome shotgun fragment and paired-end sequencing libraries (3 kb and 8 kb) were constructed from the gDNAs, as described [39, 56], and sequenced on the Illumina HiSeq2000 platform.

Linker and adapter sequences were trimmed, and cleaned reads were assembled using ALLPATHS-LG [57]. Pygap, an in-house assembly improvement tool, was used to join and extend contigs using unassembled reads when possible. Annotation of different features present in the assembly was done as previously described [58] and outlined in S1 Text.

Total RNA was extracted from eggs and adults from the gall bladder of naturally infected sheep and metacercariae (Baldwin Aquatics Inc., Monmouth, Oregon) using TRIzol reagent (Invitrogen/Life Technologies, Carlsbad, CA) according to the manufacturer’s instructions, and treated with Ambion Turbo DNase (Ambion/Applied Biosystems, Austin, TX). As previously described [59], RNA quality and yield were assessed, the purified RNA was poly(A) selected, reverse transcribed, paired-end cDNA libraries were generated, sequenced on the Illumina HiSeq 2000 platform and reads were analytically processed. Remaining, high-quality RNAseq reads (from one egg, two metacercariae and two adult biological replicates) were aligned to the genome assembly and constitutively expressed and differentially expressed genes were identified using standard protocols outlined in S1 Text.

### Identification, assembly and annotation of the genome of *Neorickettsia* from *F*. *hepatica* Oregon, isolate *nFh*

A total of 126 contigs were identified as being from bacterial origin in the *F*. *hepatica* genome assembly, and BLAST analyses indicated significant homology to *Neorickettsia* species. The total complement of raw reads were re-mapped to the 126 *Neorickettsia* contigs using BWA-MEM version 7.10 with default parameters [60], and matching reads were assembled and assembly improved using standard protocols (*see* S1 Text). The genome assembly was annotated via the NCBI prokaryotic genome annotation pipeline [61].

### Functional annotation of deduced proteins of *F*. *hepatica* and *nFh*, and MultiParanoid analyses

Deduced protein sequences were subjected to BLASTP against informative databases, including NCBI NR, InterPro, gene ontology (GO), KEGG, MEROPS using default cutoffs and release versions as specified in the S1 Text. Module completion was assessed as described [39], and transmembrane domains and classical secretion peptides were predicted using standard protocols (*see* S1 Text).

Inferred protein sequences of *F*. *hepatica* were compared to proteins from other trematodes & cognate mammalian hosts and from *Neorickettsia* were compared to proteins from representative species from the Anaphasmataceae, included all four fully sequenced *Neorickettsia* (accession numbers are provided in S1 Text). Orthologous protein families (OPFs) were constructed from pairwise InParanoid comparisons using MultiParanoid [62]. More detailed phylogenetic analyses at a level of rRNA sequences or single copy genes were performed as outlined in the S1 Text. *F*. *hepatica* Oregon and *F*. *hepatica* UK gene sets were compared using orthologs identified by Orthofinder v. 0.7.1 [63].

### Genetic variation in *Neorickettsia* and *F*. *hepatica* nuclear genomes

We sequenced the genomic DNA of an *F*. *hepatica* isolate from Uruguay that was PCR-positive for *Neorickettsia* using the Illumina platform (2 × 100bp paired-end sequencing), as previously described [56]. We included the published genomes of the United Kingdom isolates (SRA Project ID: ERP006249) in the variant analysis to help contextualize our data. Genomic reads were mapped against the combined Oregon reference assembly of *Neorickettsia and F*. *hepatica* using bwa v0.7.15 [60], followed by removal of PCR and optical duplicates using picard tools v2.6.0 [64]. Single-nucleotide variants were called via local de-novo assembly of haplotypes using the GATK pipeline v3.6 [65]. The following set of quality filters were applied to obtain high-confidence SNP calls: DP (maximum depth) > median depth+(median absolute deviation×1.4826)×2; QD (variant confidence divided by the unfiltered depth of non-reference samples) < 2.0; FS (Phred-scaled p-value using Fisher’s Exact Test to detect strand bias in the reads) > 60.0; MQ (Root Mean Square of the mapping quality of the reads across all samples) < 40.0; MQRankSum (Mann-Whitney Rank Sum Test for mapping qualities) < -12.5; ReadPosRankSum (Mann-Whitney Rank Sum Test for the distance from the end of the read for reads with the alternate allele) < -8.0. Using SnpEff [66], variants were annotated based on their genomic locations and predicted coding effects. The genetic distance between isolates (1-ibs, identity by state) were computed using PLINK v1.90 after excluding loci with missing genotypes in any of the isolates.

### Detection of *Neorickettsia* by PCR

To investigate vertical transmission of *nFh*, genomic DNA was extracted from individual worms obtained after two experimental infections in bovines (*Bos taurus*) performed at the Experimental Farm of the Institute of Hygiene, Montevideo, Uruguay, following international standards for care of research animals, and approved by the National Committee of Experimental Animal Health (CHEA). Polled Hereford calves negative to *F*. *hepatica* by fecal egg count and ELISA (Piacenza et al., 1999) and treated orally with ivemectin 1% (Mexiver, Laboratorios Santa Elena), were used in immunization studies that included challenge infection by mouth with 400 metacercariae (MCs). The MCs were obtained from Baldwin Aquatics, Oregon (assay 1) or DILAVE, Uruguay (assay 2). The cattle were euthanized at a commercial abattoir on week 20, and adult flukes were recovered from the liver of each of the calves, and flukes from each calf stored separately in >70% ethanol. Liver fluke eggs from the gall bladder of the calves also were recovered, and stored in the immunization group. DNA was extracted from individual worms of each assay, and from the pooled eggs, as described above. The presence of *Neorickettsia* within the *F*. *hepatica* adult flukes was investigated by nested PCR directed to the 16s rRNA gene, as described [67].

### Histological examination of *F*. *hepatica* and its *Neorickettsia* endobacterium

Oregon strain flukes from sheep (and *Neorickettsia*-negative flukes from Uruguay) were fixed first in 70% ethanol and then in 10% buffered formalin overnight, tissue processed (Shandon 1000 Tissue Processor, Thermo Scientific, Waltham, MA, USA), embedded in paraffin, sectioned at 5 μm. Serial sections were used for immunohistochemical studies and hematoxylin & eosin staining (S6 Fig). The sections stained with hematoxylin & eosin (according to standard technique) were used to assess morphology and determine the anatomical structures to be expected on adjacent slides used for immunohistochemical localization of *nFh*. Unstained tissue sections were rehydrated and blocked with 5% bovine serum albumin (Sigma, St. Louis MO, USA) for 30 min to prevent non-specific antibody binding. Polyclonal mouse antisera raised against a recombinant *Neorickettsia* surface protein from *Plagiorchis elegans* (Genbank Accession KX082665, *PeN*sp-3) diluted 1:250 in phosphate buffered saline containing 0.1% Triton-X and 1% bovine serum albumin was used as the primary antibody. Anti-mouse IgG Alexa Fluor 488 (Invitrogen) was used as a secondary antibody for fluorescence microscopy. Wheat germ agglutinin 633 (200 μg/ml, Invitrogen, Carlsbad, CA, USA) and DAPI (Prolong Antifade with DAPI, Molecular Probes by Life Technologies, Carlsbad, CA, USA) were used to label membranes and double-stranded DNA, respectively. Sections were examined using a wide field fluorescence microscope (WFFM, Zeiss Axios Imager Upright Fluorescence Microscope) with plan-apochromat 100X oil, 63X or 40X objectives. Fluorescence microscopy was performed at the Washington University Molecular Microbiology Imaging Facility (http://micro.imaging.wustl.edu/).

## Supporting Information

S1 Fig A comparison of the protein coding genes of *Fasciola hepatica* Oregon and *Fasciola hepatica* UK. (A) Histogram indicates the size distribution of the predicted protein coding sequences of both *Fasciola* genomes. *F*. *hepatica* UK contains an abundance of very short genes (as small as 3bp) but more large (>600bp) genes. While the proteins predicted from the two genomes do not correspond well with one another (B), functional elements appear to be shared (C). KEGG Orthologous groups (KO) shared among the *F*. *hepatica* genomes.
(PDF)

S2 Fig Gene expression information for *Fasciola hepatica*. Gene expression was profiled in eggs, metacercariae (MC), and sexually mature adults (hermaphrodites). (A) Clustering of samples based on gene expression (fragments per kilobase per million reads mapped, FPKM) indicated that eggs were more closely related to adults (which, themselves, contain eggs) than to metacercariae. (B) Differential expression of *F*. *hepatica* genes between the diverse egg, metacercariae and adult life cycle stages. Differentially expressed genes were significantly more likely than other genes to be phylogenetically conserved across all species test (P = 0.006) and more likely to contain transmembrane domains (P = 0.015). Genes with higher expression in metacercariae were enriched for several GO terms related to signal transduction and organismal development (S4 Table), and were less likely to be conserved with other FBTs (P = 1 x 10^−7^), which is not surprising given that *F*. *hepatica* metacercariae encyst on plants rather than within fish or crustaceans. In contrast, the 3,811 genes upregulated in adult flukes were enriched for microtubule based movement, redox regulation, and metabolic processes (S4 Table), as previously found in expression studies of *F*. *hepatica* from the UK [68]. The genes overexpressed in adults compared to metacercariae were more likely to be FBT conserved and specific (P = 2 x 10^−7^) and to have orthologs in mammals but not the free-living platyhelminth *S*. *mediterranea* (P = 6 x 10^−10^), suggesting potential roles in host interaction. (C) Summary of Illumina RNAseq reads, and SRA accessions.
(TIF)

S3 Fig Metabolic and catabolic liver fluke pathway details. (A) Fatty acid elongation by reversal of beta-oxidation (B) Fatty acid degradation (C) Aliphatic amino acid catabolism. Enzymes present in *F*. *hepatica* are identified in yellow, and those present in *S*. *mansoni* in blue. Image generated with the KEGGscan_pathway at trematode.net (http://trematode.net).
(TIF)

S4 Fig Clustering of species and modules. Clustering based on complete (A) and lenient (B) completion of KEGG metabolic pathways modules in each species (light green is “incomplete with < 3 reaction steps. Modules with 2 steps have been manually filled in after combining “strict” and “lenient” results). Oa = *Ovis aries*, Bt = *Bos taurus*, Hs = *Homo sapiens*, FhOREGON = *F*. *hepatica*, Oregon strain, FhUK = *F*. *hepatica*, UK strain, Sm = *Schistosoma mansoni*, Cs = *Clonorchis sinensis*, Ov = *Opisthorchis viverrini*.
(TIF)

S5 Fig PCR analysis for *Neorickettsia* from diverse isolates of *Fasciola hepatica*. Nested PCR for the bacterial 16s RNA gene in five different *F*. *hepatica* flukes from Uruguay (lanes 3–7) and the reference Oregon strain (8). nF*h*-positive signals were observed in one sample from Uruguay and the Oregon isolate.
(TIF)

S6 Fig Examples of standard H&E stain of sections of adult *F*. *hepatica* to help identify organs and tissues examined for localization of *Neorickettsia*. (A) Cross-section of the proximal part of *F*. *hepatica*. (B) Cross-section of the distal part of *F. hepatica*. (C) Longitudinal section of *F*. *hepatica*. Bar corresponds to 1 mm.
(TIF)

S7 Fig Detection of nFh 16s rRNA gene by PCR. Primary (top panel) and secondary (nested) PCR for the bacterial 16s RNA gene following protocol previously described [65]. DNA from *nFh*-negative sample from Uruguay (lane 11) and *nFh*-positive sample from Oregon (lane 13). To further test if the bacteria might have been transmitted through the parasite, we tested by PCR individual flukes isolated after two different experimental infections performed with metacercariae from Uruguay (lanes 3–10) and Oregon (lanes 16–23) respectively, and eggs collected from these experimental infections (lanes 1–2, and 14–15). Since nested PCR was performed using dilution of primary amplicons without band purification, carry over of first round primers occurred, visible as a doublet band below the primary amplification, corresponding to the expected nested product (lower band) and byproducts between the external and internal primers. The identity of the *nFh* 16s rRNA gene was confirmed by nucleotide sequencing.
(TIF)

S8 Fig Localization of *Neorickettsia* in male reproductive tissue of *F*. *hepatica* Oregon. (A) Cross-section of the vas deferens with overlay of the individual stains for plasma membranes (wheat germ agglutinin, WGA), double stranded DNA (DAPI) and *Neorickettsia* (Nsp). (B) Individual stain for plasma membranes (red). (C) Individual stain for DNA. Note the strong blue stain of spermatozoa and the lighter bluish stain of low DNA content *Neorickettsia* (arrows). D. Individual green stain for *Neorickettsia* (arrows). Bar corresponds to 100 μm.
(TIF)

S1 Table The nuclear genome assemblies of *F*. *hepatica* Oregon and other food borne trematodes. (DOCX)

S2 Table Repetitive elements, tRNA and rRNA in the genome of *Fasciola hepatica* Oregon. (DOCX)

S3 Table *F*. *hepatica* genome summary table. (XLSX)

S4 Table Functional enrichment among *F*. *hepatica* gene sets of interest. (XLSX)

S5 Table *Neorickettsia F*. *hepatica* genome summary table. (XLSX)

S6 Table Coverage statistics for *Fasciola hepatica* and *Neorickettsia*. (DOCX)

S7 Table Non-synonymous SNPs between the *Neorickettsia* genomes of *Fasciola hepatica* Oregon (US) and Uruguay (UY) isolates. (DOCX)

S8 Table Genetic distance (1-ibs, identity by state) between *Fasciola hepatica* isolates based on the nuclear SNPs. (DOCX)

S1 Text Supporting materials and methods. (DOCX)
