# Supplementary figures and images for: Genomes of Fasciola hepatica from the Americas Reveal Colonization with Neorickettsia Endobacteria Related to the Agents of Potomac Horse and Human Sennetsu Fevers
Source: PLoS Genet. 2017 Jan 6;13(1):e1006537. doi: 10.1371/journal.pgen.1006537 (PMC5257007; doi:10.1371/journal.pgen.1006537)

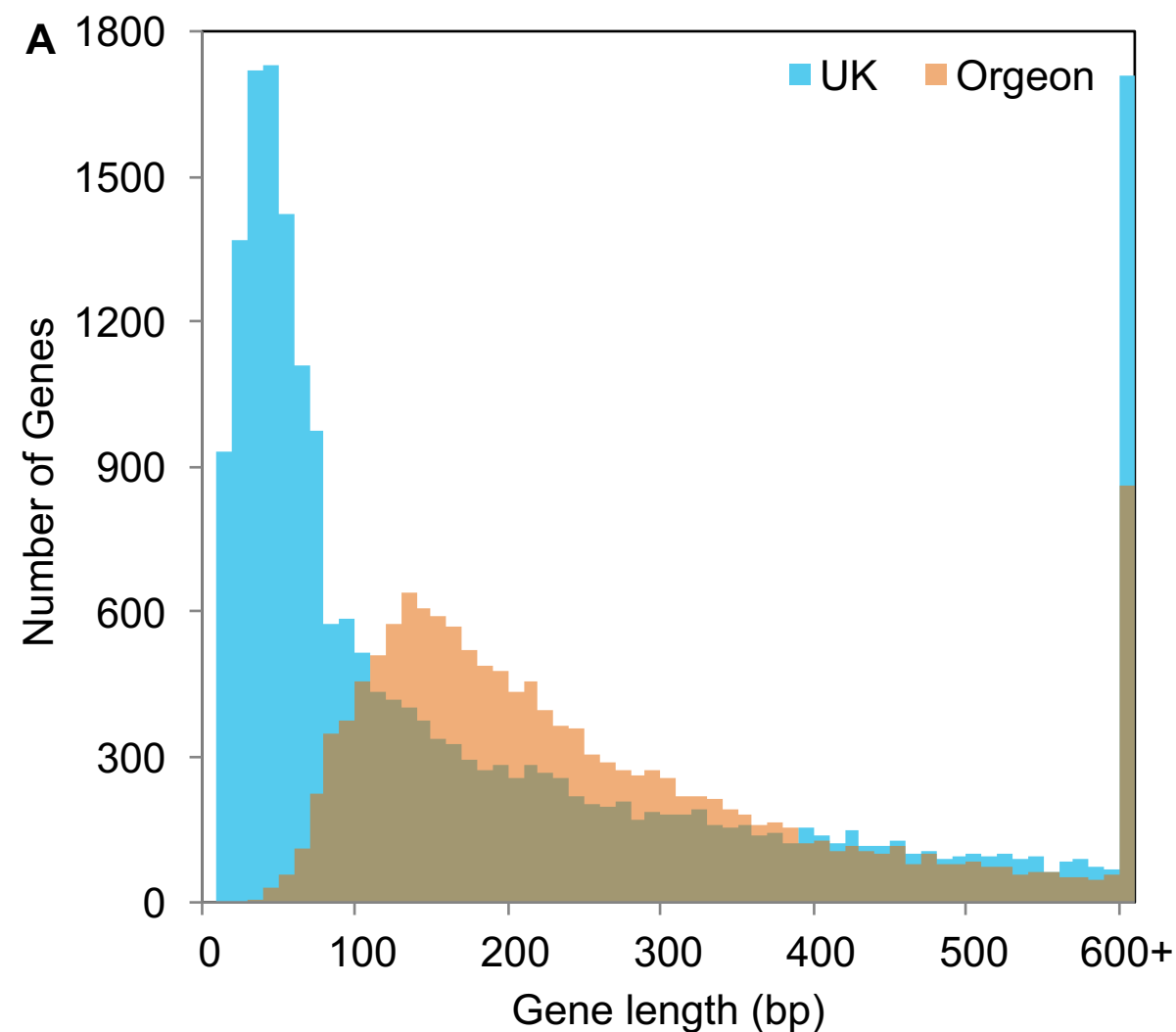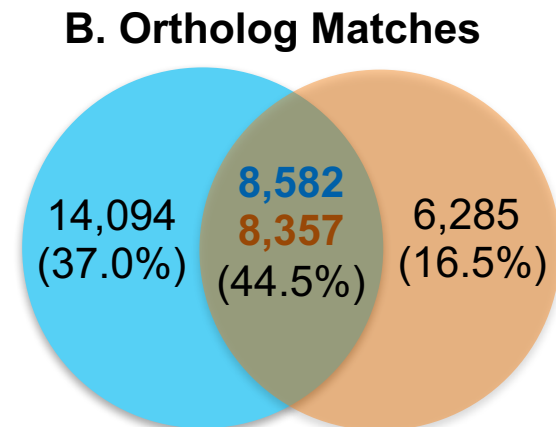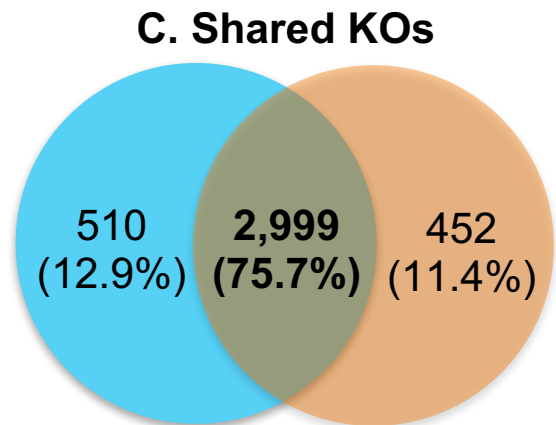

Supplement: S1 Fig — (A) Histogram indicates the size distribution of the predicted protein coding sequences of both Fasciola genomes. F. hepatica UK contains an abundance of very short genes (as small as 3bp) but more large (>600bp) genes. While the proteins predicted from the two genomes do not correspond well with one another (B), functional elements appear to be shared (C). KEGG Orthologous groups (KO) shared among the F. hepatica genomes. (PDF) [file pgen.1006537.s001.pdf]

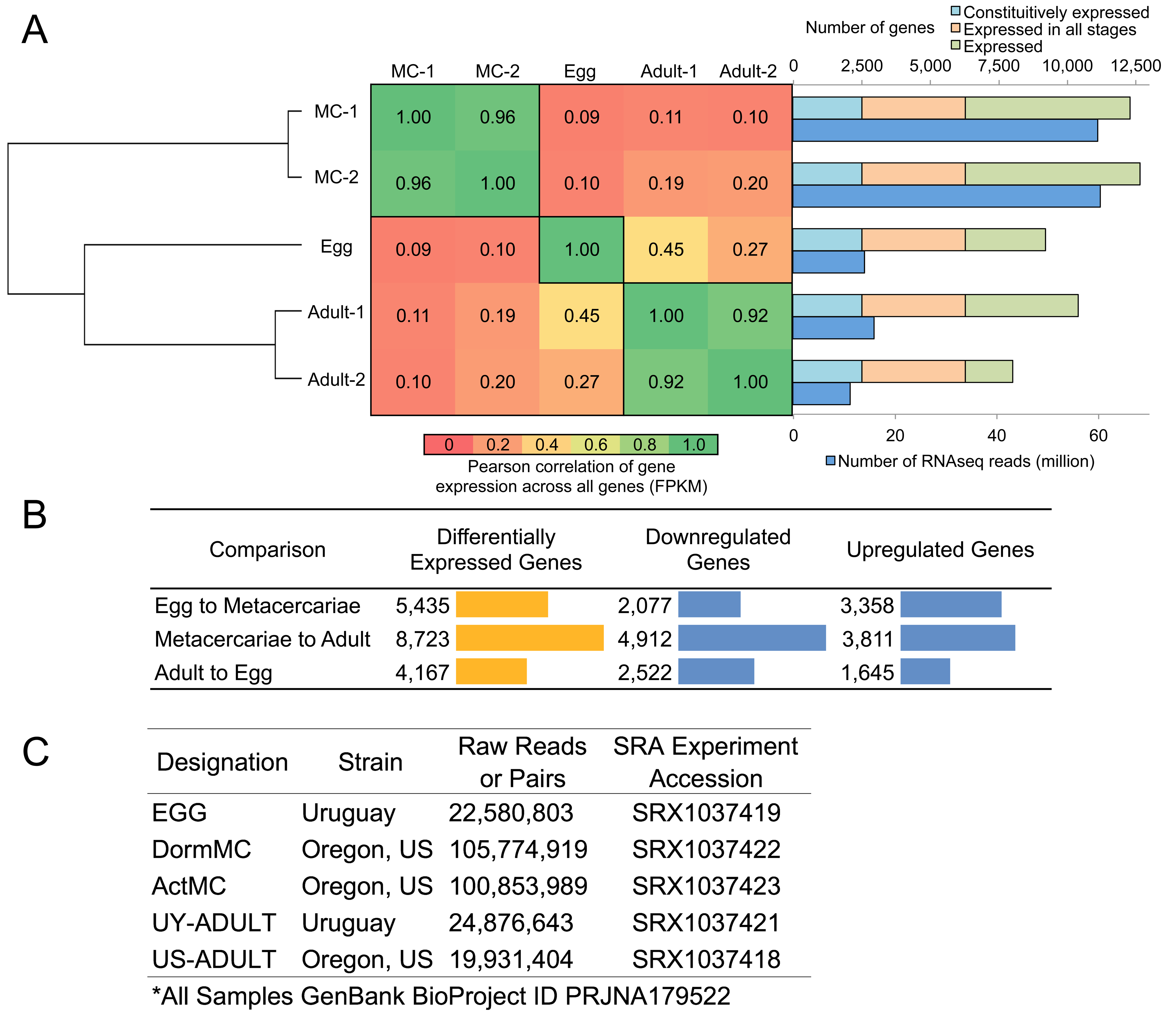

Supplement: S2 Fig — Gene expression was profiled in eggs, metacercariae (MC), and sexually mature adults (hermaphrodites). (A) Clustering of samples based on gene expression (fragments per kilobase per million reads mapped, FPKM) indicated that eggs were more closely related to adults (which, themselves, contain eggs) than to metacercariae. (B) Differential expression of F. hepatica genes between the diverse egg, metacercariae and adult life cycle stages. Differentially expressed genes were significantly more likely than other genes to be phylogenetically conserved across all species test (P = 0.006) and more likely to contain transmembrane domains (P = 0.015). Genes with higher expression in metacercariae were enriched for several GO terms related to signal transduction and organismal development (S4 Table), and were less likely to be conserved with other FBTs (P = 1 x 10^−7^), which is not surprising given that F. hepatica metacercariae encyst on plants rather than within fish or crustaceans. In contrast, the 3,811 genes upregulated in adult flukes were enriched for microtubule based movement, redox regulation, and metabolic processes (S4 Table), as previously found in expression studies of F. hepatica from the UK [68]. The genes overexpressed in adults compared to metacercariae were more likely to be FBT conserved and specific (P = 2 x 10^−7^) and to have orthologs in mammals but not the free-living platyhelminth S. mediterranea (P = 6 x 10^−10^), suggesting potential roles in host interaction. (C) Summary of Illumina RNAseq reads, and SRA accessions. (TIF) [file pgen.1006537.s002.tif]

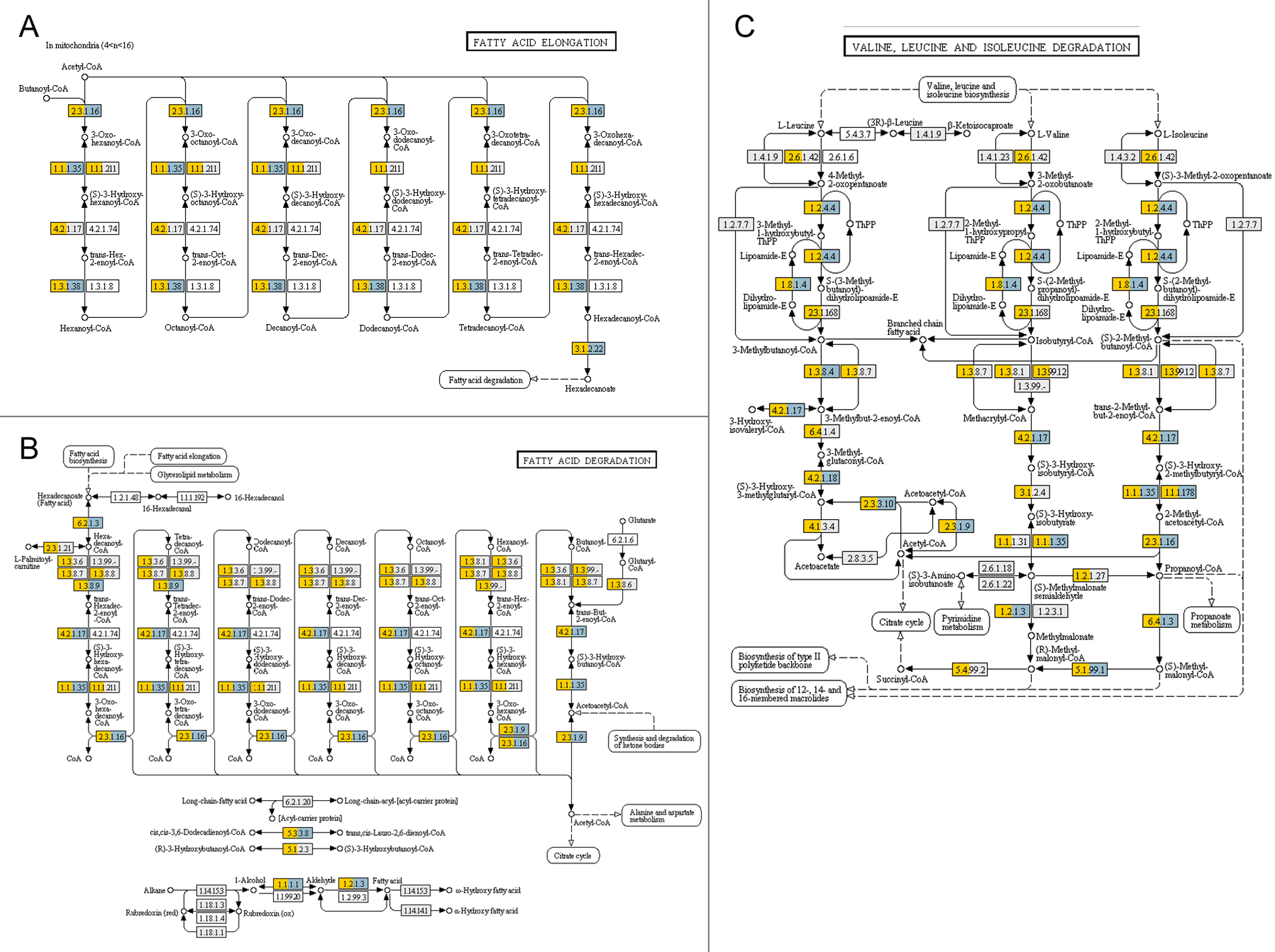

Supplement: S3 Fig — (A) Fatty acid elongation by reversal of beta-oxidation (B) Fatty acid degradation (C) Aliphatic amino acid catabolism. Enzymes present in F. hepatica are identified in yellow, and those present in S. mansoni in blue. Image generated with the KEGGscan_pathway at trematode.net (http://trematode.net). (TIF) [file pgen.1006537.s003.tif]

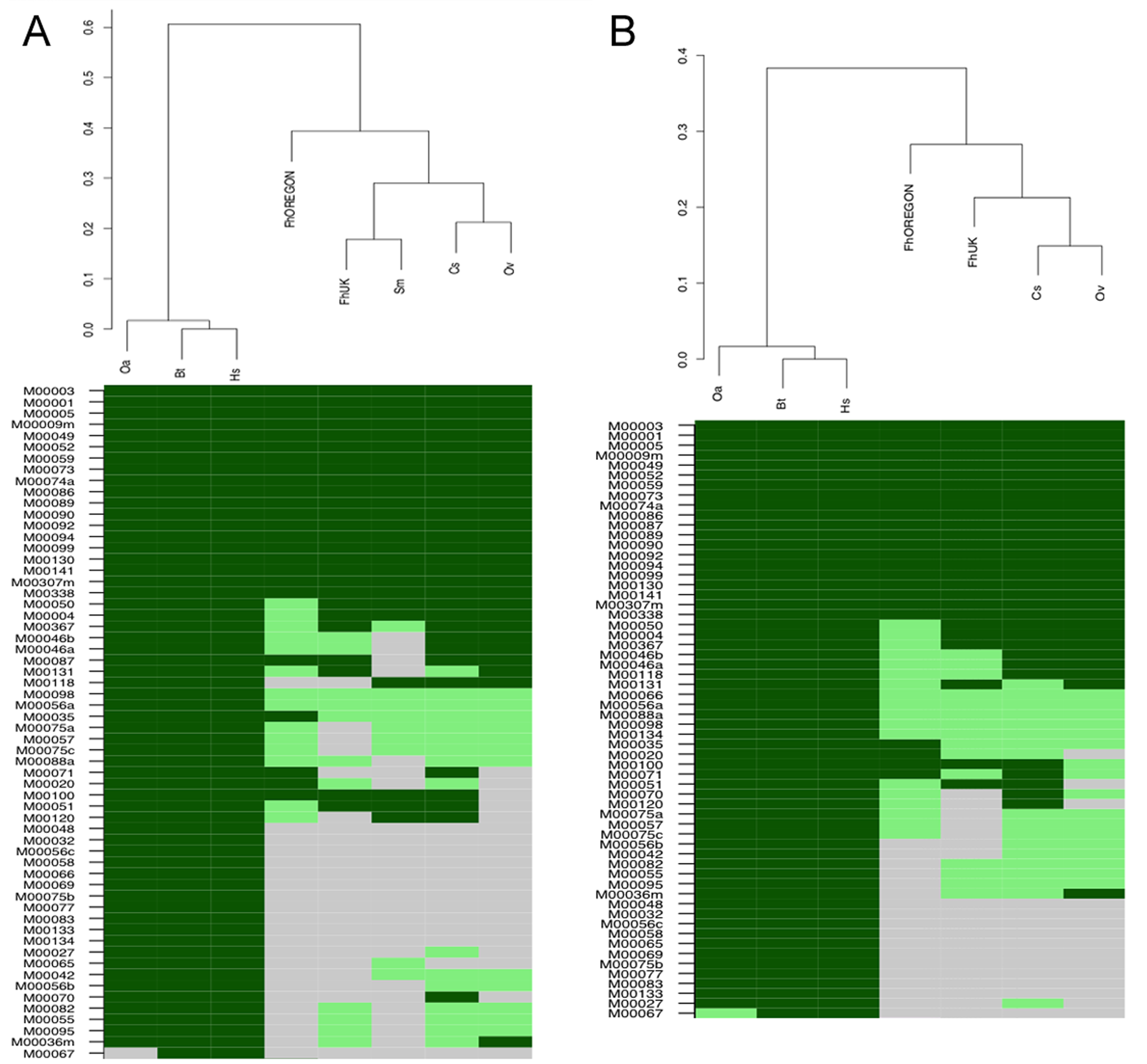

Supplement: S4 Fig — Clustering based on complete (A) and lenient (B) completion of KEGG metabolic pathways modules in each species (light green is “incomplete with < 3 reaction steps. Modules with 2 steps have been manually filled in after combining “strict” and “lenient” results). Oa = Ovis aries, Bt = Bos taurus, Hs = Homo sapiens, FhOREGON = F. hepatica, Oregon strain, FhUK = F. hepatica, UK strain, Sm = Schistosoma mansoni, Cs = Clonorchis sinensis, Ov = Opisthorchis viverrini. (TIF) [file pgen.1006537.s004.tif]

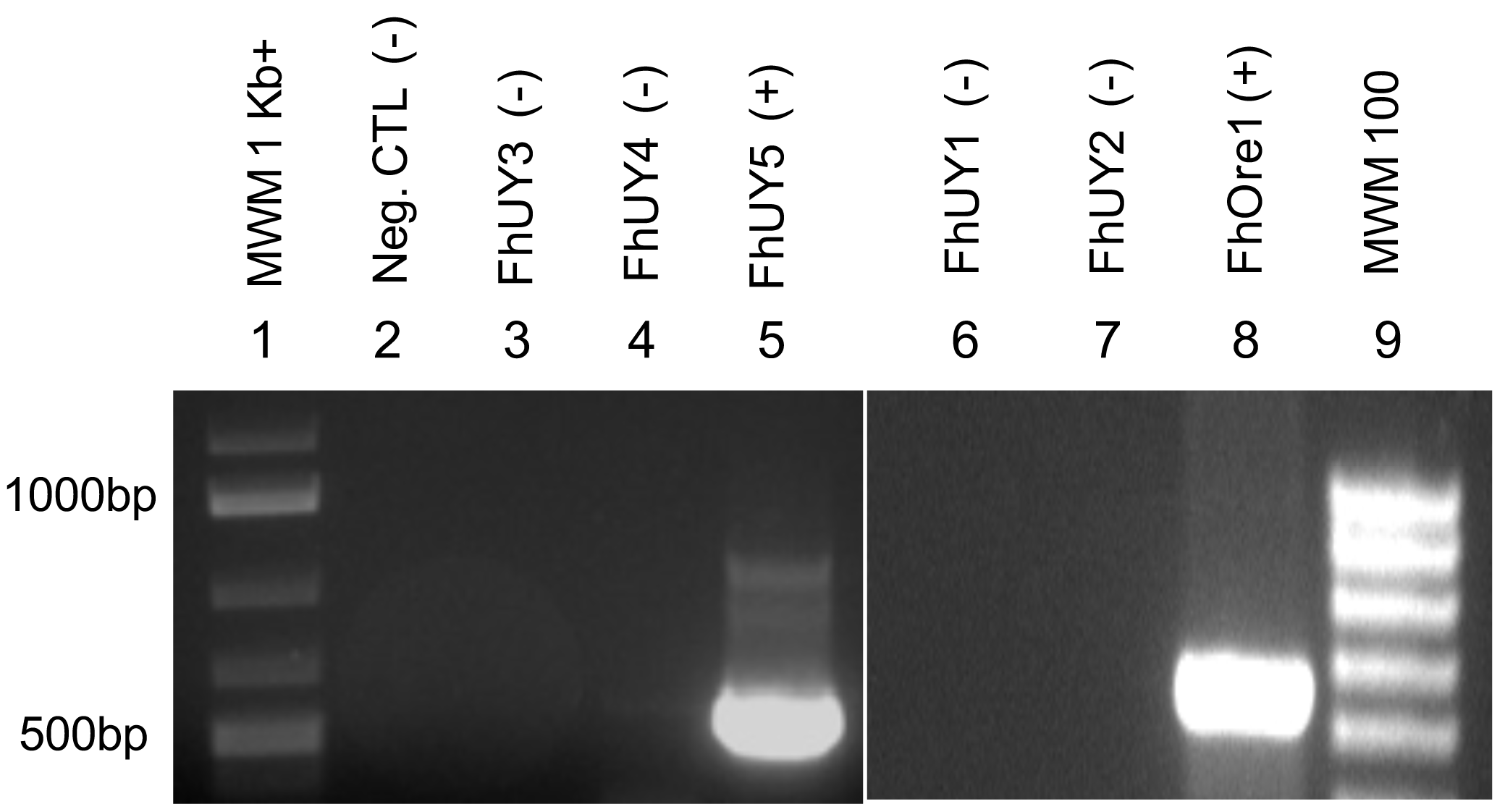

Supplement: S5 Fig — Nested PCR for the bacterial 16s RNA gene in five different F. hepatica flukes from Uruguay (lanes 3–7) and the reference Oregon strain (8). nFh-positive signals were observed in one sample from Uruguay and the Oregon isolate. (TIF) [file pgen.1006537.s005.tif]

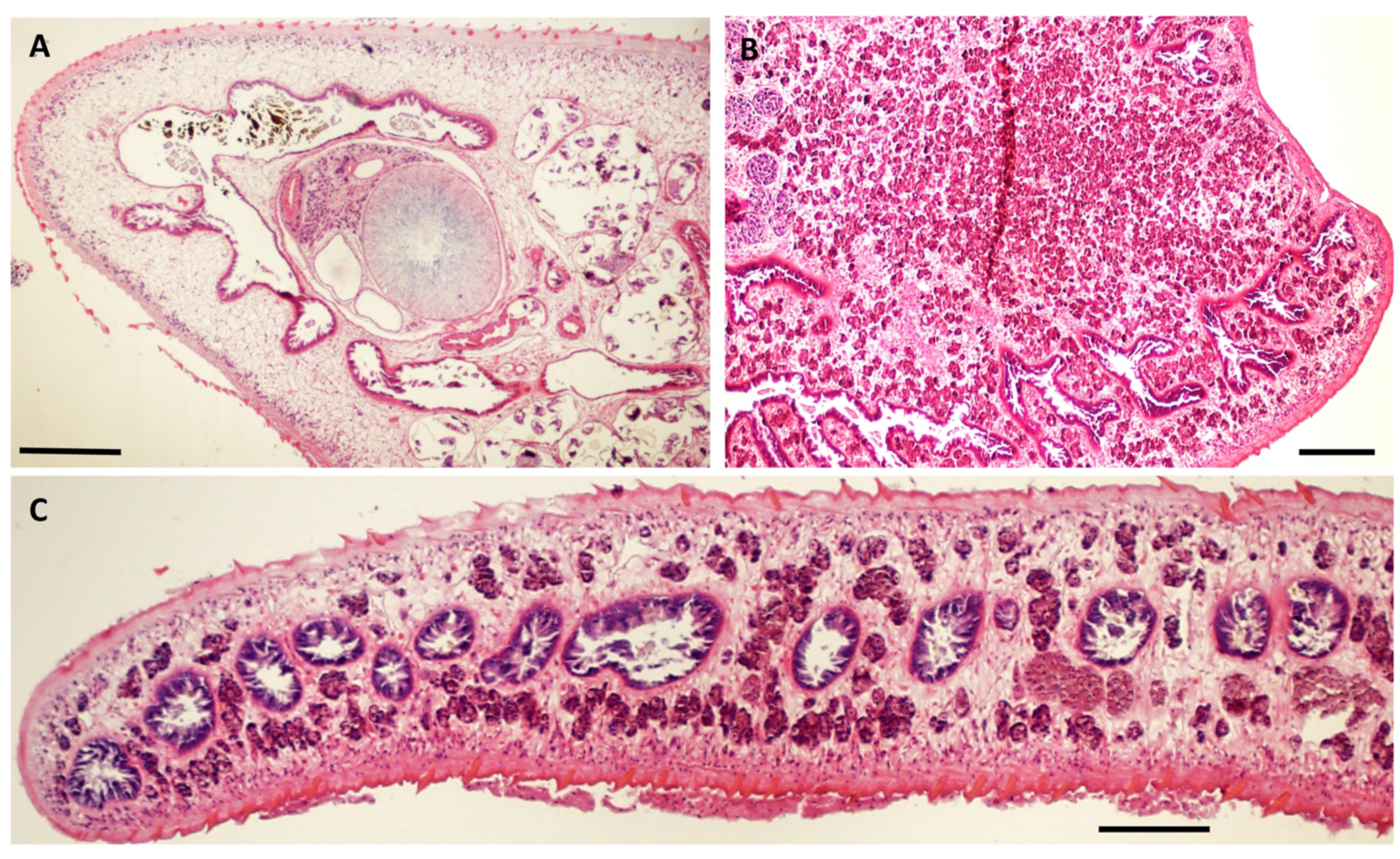

Supplement: S6 Fig — (A) Cross-section of the proximal part of F. hepatica. (B) Cross-section of the distal part of F. hepatica. (C) Longitudinal section of F. hepatica. Bar corresponds to 1 mm. (TIF) [file pgen.1006537.s006.tif]

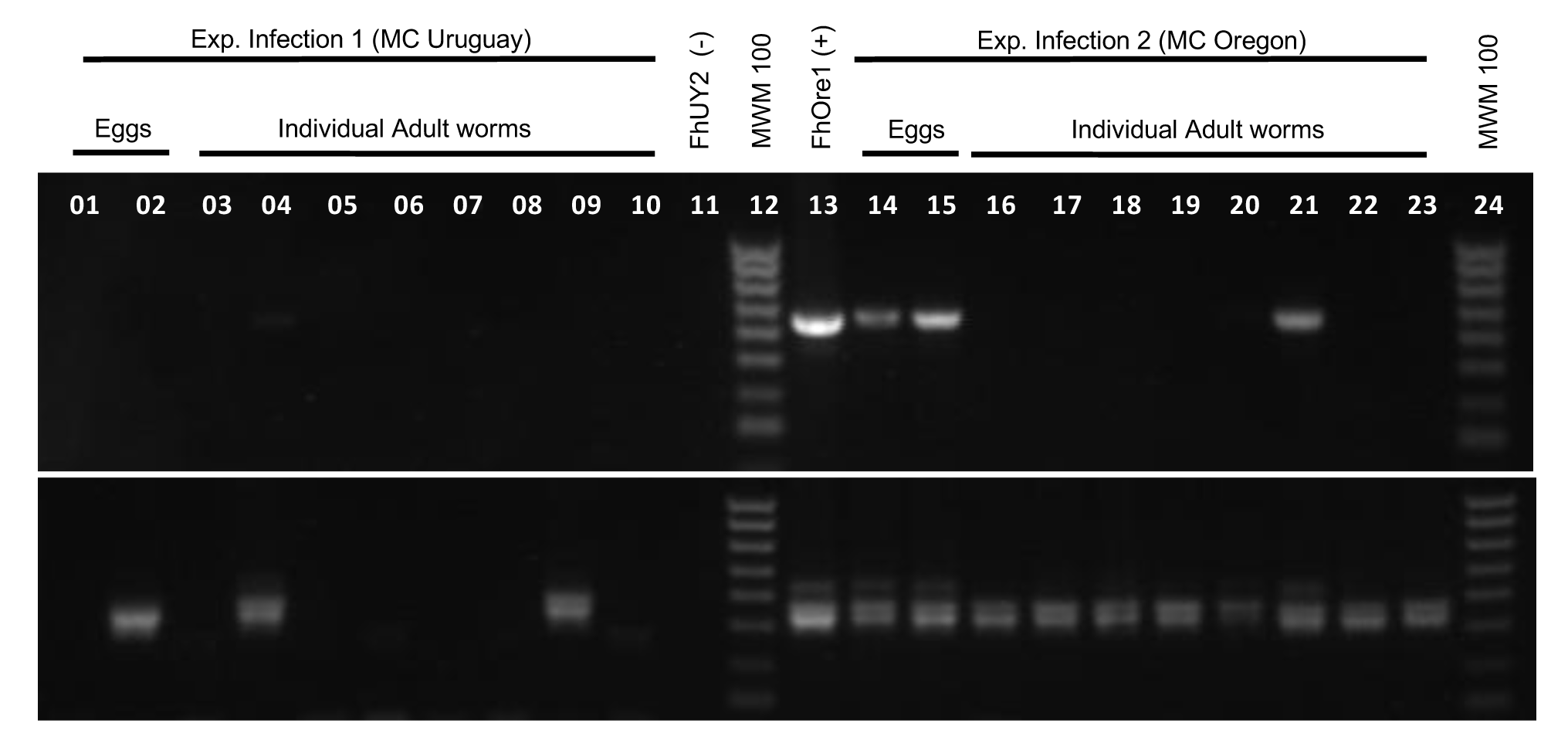

Supplement: S7 Fig — Primary (top panel) and secondary (nested) PCR for the bacterial 16s RNA gene following protocol previously described [65]. DNA from nFh-negative sample from Uruguay (lane 11) and nFh-positive sample from Oregon (lane 13). To further test if the bacteria might have been transmitted through the parasite, we tested by PCR individual flukes isolated after two different experimental infections performed with metacercariae from Uruguay (lanes 3–10) and Oregon (lanes 16–23) respectively, and eggs collected from these experimental infections (lanes 1–2, and 14–15). Since nested PCR was performed using dilution of primary amplicons without band purification, carry over of first round primers occurred, visible as a doublet band below the primary amplification, corresponding to the expected nested product (lower band) and byproducts between the external and internal primers. The identity of the nFh 16s rRNA gene was confirmed by nucleotide sequencing. (TIF) [file pgen.1006537.s007.tif]

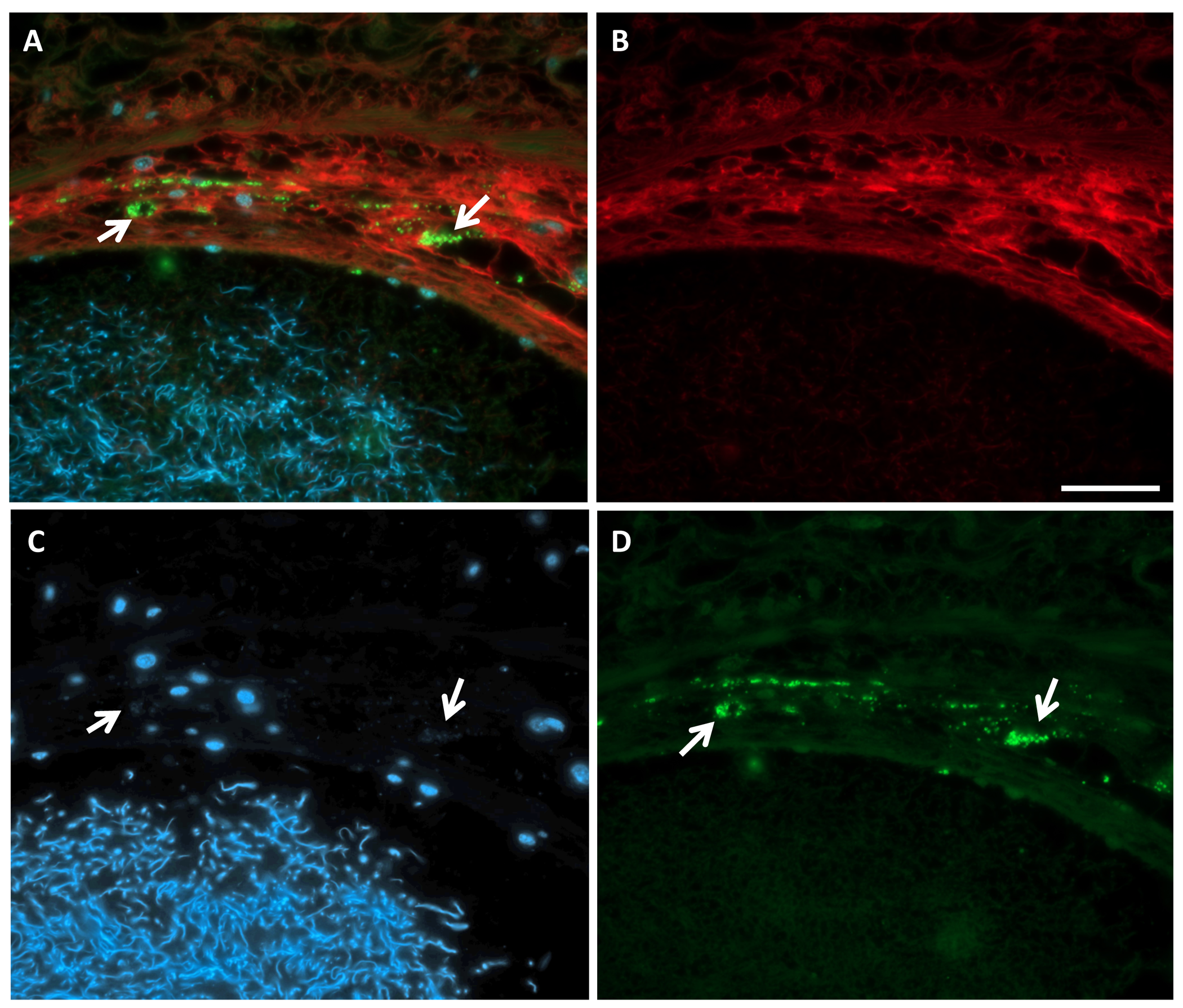

Supplement: S8 Fig — (A) Cross-section of the vas deferens with overlay of the individual stains for plasma membranes (wheat germ agglutinin, WGA), double stranded DNA (DAPI) and Neorickettsia (Nsp). (B) Individual stain for plasma membranes (red). (C) Individual stain for DNA. Note the strong blue stain of spermatozoa and the lighter bluish stain of low DNA content Neorickettsia (arrows). D. Individual green stain for Neorickettsia (arrows). Bar corresponds to 100 μm. (TIF) [file pgen.1006537.s008.tif]
