# Supplementary material for: Genomes of Fasciola hepatica from the Americas Reveal Colonization with Neorickettsia Endobacteria Related to the Agents of Potomac Horse and Human Sennetsu Fevers
Source: PLoS Genet. 2017 Jan 6;13(1):e1006537. doi: 10.1371/journal.pgen.1006537 (PMC5257007; doi:10.1371/journal.pgen.1006537)
Supplement: S1 Table — (DOCX) [file pgen.1006537.s009.docx]

## S1 Table. The nuclear genome assemblies of *F. hepatica* Oregon and other food borne trematodes.

|  | ***F. hepatica* Oregon**** | ***F. hepatica***  **UK** | ***Clonorchis sinensis*** | ***Opisthorchis viverrini*** |
| --- | --- | --- | --- | --- |
| Source | This study | (Cwiklinski et al. 2015) | (Huang et al. 2013) | (Young et al. 2014) |
| Assembly Length | 1,138,330,036 bp | 1,275,107,963 bp | 547,288,241 bp | 620,452,578 bp |
| Length of inferred gaps (N’s) | 58,314,589  bp | 91,603,602  bp | 184,200  bp | 48,341,148 bp |
| Contigs | 23,605* | 20,158 | 4,348 | 42,216 |
| Assembly N50 (number/length) | 2,036 /  161,103 bp | 1,799 / 204,014 bp | 408 / 417,486 bp | 138 / 1,349,843 bp |
| GC content | 44.1% | 44.1% | 44.1% | 43.8% |
| Repetitive sequences | 55.3% | 32.0% | 25.6% | 30.6% |
| Protein coding genes | 14,642 | 22,676 | 13,634 | 16,356 |
| Average CDS length | 837 bp | 773 bp*** | 1,591 bp | 1,301 bp |
| Minimum CDS | 81 bp | 3 bp | 150 bp | 90 bp |
| Maximum CDS | 13,770 bp | 25,797 bp | 48,063 bp | 32,826 bp |
| % coding | 1.08% | 1.49% | 3.89% | 3.42% |
| Average gene footprint | 7,286 bp | 13,713 bp | 17,760 bp | 18,221 bp |
| Single exon genes | 2,188 | 576 | 1,716 | 2,438 |
| Multi-exon genes | 10,140 | 22,100 | 11,918 | 13,918 |
| Average exons per multi-exon gene | 3.3 | 5.3 | 7.7 | 6.6 |
| Average exon size | 259.8 bp | 288.9 bp | 231.7 bp | 253.1 bp |

*Includes scaffolds (contigs joined by inferred gaps) and singleton contigs >1kb.

**Gapped genes (i.e., genes called across multiple contigs) were excluded from length and intron/exon calculations

***Calculated from longest isoform of each protein-coding gene
