# Supplementary material for: Genomes of Fasciola hepatica from the Americas Reveal Colonization with Neorickettsia Endobacteria Related to the Agents of Potomac Horse and Human Sennetsu Fevers
Source: PLoS Genet. 2017 Jan 6;13(1):e1006537. doi: 10.1371/journal.pgen.1006537 (PMC5257007; doi:10.1371/journal.pgen.1006537)
Supplement: S2 Table — (DOCX) [file pgen.1006537.s010.docx]

## S2 Table. Repetitive elements, tRNA and rRNA in the genome of *Fasciola hepatica* Oregon.

| **Repeat** | Number of Elements | Length Occupied (bp) | Percentage of Sequence |
| --- | --- | --- | --- |
| SINEs | 65,062 | 11,712,840 | 1.03% |
| ALUs | 0 | 0 | 0.00% |
| MIR | 0 | 0 | 0.00% |
| LINEs | 458,935 | 268,097,580 | 23.55% |
| LINE1 | 0 | 0 | 0.00% |
| LINE2 | 5,711 | 5,711 | 0.19% |
| L3/CR1 | 175,730 | 123,776,963 | 10.87% |
| LTR elements | 122,173 | 92,433,551 | 8.12% |
| ERVL | 0 | 0 | 0.00% |
| ERVL-MaLRs | 0 | 0 | 0.00% |
| ERV_classI | 450 | 218,266 | 0.02% |
| ERV_classII | 0 | 0 | 0.00% |
| DNA elements | 54,748 | 21,458,265 | 1.89% |
| hAT-Charlie | 0 | 0 | 0.00% |
| TcMar-Tigger | 506 | 109,636 | 0.01% |
| Unclassified | 813,674 | 235,731,691 | 20.71% |
| Total interspersed repeats |  | 629,433,927 | 55.29% |
|  |  |  |  |
| **Functional RNA** | Number of genes | Length Occupied (bp) | Percentage of Sequence |
| tRNA | 230 | 4,393 | 0.0004% |
| rRNA | 23 | 17,284 | 0.0015% |
| miRNA | 189 | 15,121 | 0.0013% |
