# Supplementary material for: Genomes of Fasciola hepatica from the Americas Reveal Colonization with Neorickettsia Endobacteria Related to the Agents of Potomac Horse and Human Sennetsu Fevers
Source: PLoS Genet. 2017 Jan 6;13(1):e1006537. doi: 10.1371/journal.pgen.1006537 (PMC5257007; doi:10.1371/journal.pgen.1006537)
Supplement: S6 Table — (DOCX) [file pgen.1006537.s014.docx]

**S6 Table.** Coverage statistics for *Fasciola hepatica* and *Neorickettsia.*

| Sample ID | *F. hepatica* (nuclear) | | *Neorickettsia* | |
| --- | --- | --- | --- | --- |
|  | Mean depth of coverage | Breadth of coverage (%) | Mean depth of coverage | Breadth of coverage (%) |
| US* | 28.24 | 98.3 | 162.54 | 99.9 |
| UY | 63.28 | 97.6 | 20.8 | 99.9 |
| UK1 | 20.72 | 96.5 | 0 | 0.1 |
| UK2 | 27.58 | 96.9 | 0 | 0.2 |
| UK3 | 17.87 | 96.7 | 0 | 0.2 |
| UK4 | 15.97 | 96.5 | 0 | 0.1 |
| UK5 | 17.25 | 96.6 | 0 | 0.1 |

* Coverage statistics were based only on small insert library reads (excluding mate-pair reads)
