# Supplementary material for: Genomes of Fasciola hepatica from the Americas Reveal Colonization with Neorickettsia Endobacteria Related to the Agents of Potomac Horse and Human Sennetsu Fevers
Source: PLoS Genet. 2017 Jan 6;13(1):e1006537. doi: 10.1371/journal.pgen.1006537 (PMC5257007; doi:10.1371/journal.pgen.1006537)
Supplement: S7 Table — (DOCX) [file pgen.1006537.s015.docx]

**S7 Table.** Non-synonymous SNPs between the *Neorickettsia* genomes of *Fasciola hepatica* Oregon (US) and Uruguay (UY) isolates.

| Position | Ref allele  (US) | Alt allele  (UY) | Protein change | Gene ID | Gene description |
| --- | --- | --- | --- | --- | --- |
| 85000 | C | A | His60Gln | AS219_00390 | Hypothetical protein |
| 143570 | A | G | Tyr73Cys | AS219_00645 | 2-C-methyl-D-erythritol 4-phosphate cytidylyltransferase |
| 165945 | A | G | Glu270Gly | AS219_00725 | DNA recombination protein RecF |
| 282693 | T | C | Ile236Val | AS219_01380 | DNA-binding protein |
| 501011 | C | T | Gly316Ser | AS219_02325 | 2-oxoglutarate dehydrogenase |
| 506569 | T | C | Thr184Ala | AS219_02365 | ATP synthase F1 subunit gamma |
| 689258 | C | T | Gly159Glu | AS219_03145 | Molybdopterin biosynthesis protein MoeB |
