# Supplementary material for: Genomes of Fasciola hepatica from the Americas Reveal Colonization with Neorickettsia Endobacteria Related to the Agents of Potomac Horse and Human Sennetsu Fevers
Source: PLoS Genet. 2017 Jan 6;13(1):e1006537. doi: 10.1371/journal.pgen.1006537 (PMC5257007; doi:10.1371/journal.pgen.1006537)

**S8 Table.** Genetic distance (1-ibs, identity by state) between *Fasciola hepatica* isolates based on the nuclear SNPs. After excluding loci with missing genotypes in any of the isolates, variant and non-variant loci (n=14,775,639) observed on the top 20 longest contigs were used for ibs estimation. The genetic distance between Oregon (US) and Uruguay (UY) isolates indicated that they are not substantially more closely related to each other than either is to the five published UK isolates (UK1-5; SRA Project ID: ERP006249).


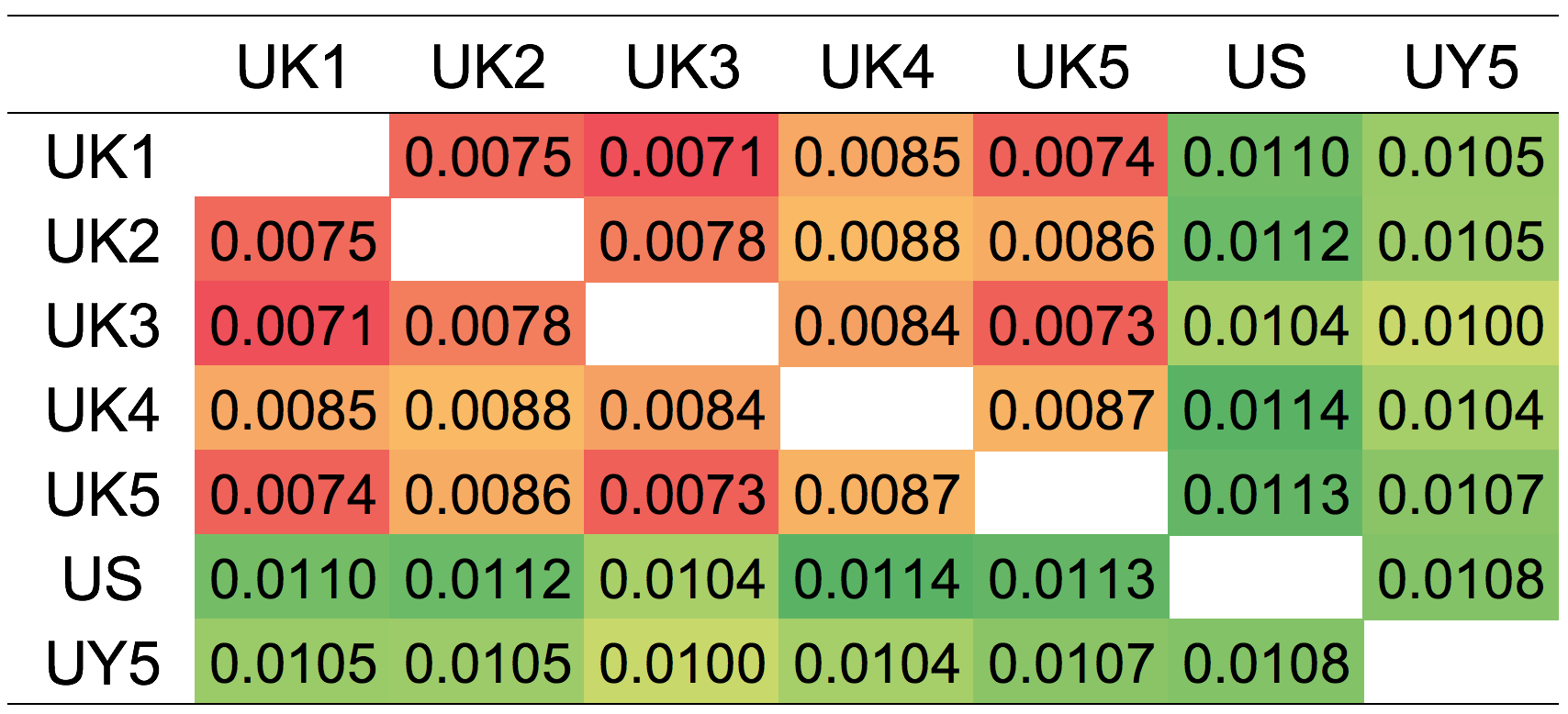

Supplement: S8 Table — (DOCX) [file pgen.1006537.s016.docx]
