# Supplementary material for: Genomes of Fasciola hepatica from the Americas Reveal Colonization with Neorickettsia Endobacteria Related to the Agents of Potomac Horse and Human Sennetsu Fevers
Source: PLoS Genet. 2017 Jan 6;13(1):e1006537. doi: 10.1371/journal.pgen.1006537 (PMC5257007; doi:10.1371/journal.pgen.1006537)
Supplement: S1 Text — (DOCX) [file pgen.1006537.s017.docx]

# Text S1: Supporting Materials and Methods

## Liver flukes

Two isolates of *Fasciola hepatica* were analyzed: worms collected from livers of naturally infected sheep from a commercial slaughterhouse in Oregon (provided by Baldwin Aquatics Inc., Monmouth, Oregon), i.e. Oregon isolate; and worms isolated from livers of naturally infected sheep obtained from a commercial slaughterhouse in Montevideo, Uruguay, i.e. Uruguay isolate. For transcriptomic analysis, total RNAs were obtained from the egg, metacercarial and adult developmental stages of *F. hepatica* (in duplicate). Eggs were collected from gall bladder of naturally infected sheep. Metacercariae were purchased from Baldwin Aquatics Inc. (Monmouth, Oregon). Tissue sections for the histological analysis were prepared from adult worms of the Oregon and Uruguay isolates.

## DNA isolation and sequencing

Genomic DNA (gDNA) was extracted using the kit E.Z.N.A. SQ Tissue DNA Kit (Omega Bio-tek). In brief, fresh or ethanol-preserved adult worms were fragmented using a scalpel blade, and lysed in the kit buffer containing detergent. Proteinase K was added, and the lysate incubated at 58**°**C for 18 hours. The digest was incubated with RNase at 37**°**C for one hour, after which proteins were precipitated. The gDNA in the supernatant was precipitated with isopropanol, after the gDNA was washed with ethanol and dissolved in hydration buffer, and the yield and purified assessed by Bo-Analyzer. Whole genome shotgun fragment and paired-end sequencing libraries (3 kb and 8 kb) were constructed from the gDNAs, as described (Tyagi et al. 2015a; McNulty et al. 2016), and sequenced on the Illumina HiSeq2000 platform.

## Assembly and annotation of the genome of *F. hepatica* Oregon isolate

Linker and adapter sequences were trimmed, and cleaned reads were assembled using ALLPATHS-LG (Gnerre et al. 2011). Pygap, an in-house assembly improvement tool, was used to join and extend contigs using unassembled reads when possible. A repeat library was generated using Repeatmodeler ([http://repeatmasker.org](http://repeatmasker.org/)), and repeats were characterized by screening against Repbase (April 2014) with CENSOR version 4.2.29 (Jurka et al. 2005; Kohany et al. 2006) (see Table S2). Ribosomal RNA genes were identified using RNAmmer (Lagesen et al. 2007), transfer RNA genes were identified using tRNAscan-SE (Lowe and Eddy 1997), and other non-coding RNAs (e.g., microRNAs) were identified by sequence homology searches against the Rfam database (Griffiths-Jones et al. 2003). miRNAs were further characterized by small RNA sequencing and mapping to the genome assembly with miRDeep2 (Friedländer et al. 2012). The resulting putative miRNAs were assigned to families by comparison with miRBase (Kozomara and Griffiths-Jones 2014). Putative predicted miRNAs were considered as novel if they find no match in miRBase and had a miRDeep2 score equal or higher than 10; the resulting predictions were then manually checked for possible false positives. Repeats and non-protein-coding RNAs were masked with RepeatMasker ([http://repeatmasker.org](http://repeatmasker.org/)).

Protein coding genes were predicted from the masked assembly using a combination of Snap (Korf 2004), Fgenesh (Salamov and Solovyev 2000), Augustus (Stanke et al. 2008), and the MAKER annotation pipeline (Cantarel et al. 2008) using evidence from assembled transcripts, ESTs (GenBank EST database), and protein sequences from *F. hepatica* and related organisms (Robinson et al. 2009; Cancela et al. 2010; Young et al. 2010; Wilson et al. 2011). A consensus gene set based on these predictions was generated using a hierarchical approach described before (Tang et al. 2014), and gene product naming was determined by BER ([http://ber.sourceforge.net](http://ber.sourceforge.net/)).

## Identification, assembly and annotation of the genome of *Neorickettsia* from *F. hepatica* Oregon, isolate *nFh*

A total of 126 contigs were identified as being from bacterial origin in the *F. hepatica* genome assembly, and BLAST analyses indicated significant homology to *Neorickettsia* species. The total complement of raw reads were re-mapped to the 126 *Neorickettsia* contigs using BWA-MEM version 7.10 with default parameters (Li and Durbin 2009), and matching reads were assembled with Velvet (version 1.1.06, (Zerbino and Birney 2008)). Contigs were assembled into scaffolds with SSPACE using all available short insert, 3kb insert, and 8kb insert reads (Boetzer et al. 2011), and gaps were filled with GapFiller using all available short insert reads (Boetzer and Pirovano 2012). The genome assembly was submitted to the NCBI Reference Sequence Database (RefSeq) for feature prediction via the NCBI prokaryotic genome annotation pipeline (Angiuoli et al. 2008). Visualization of the *Neorickettsia* genome (Figure 3) was performed using Circos v0.69 (Krzywinski et al. 2009).

## Functional annotation of deduced proteins of *F. hepatica* and *nFh*

Deduced protein sequences were subjected to BLASTP against informative databases, including NCBI non-redundant protein database ((O'Leary et al. 2016), downloaded 2015-06-05), and non-overlapping top hits to each query with e-value < 1e-^05^ were collected. Conserved protein domains (IPR domains) and gene ontology (GO) classifications were inferred using InterProScan (Ashburner et al. 2000; Quevillon et al. 2005; Hunter et al. 2012). Proteins were assigned to KEGG orthologous groups, enzyme commission classifications, biochemical pathways and pathway modules using KAAS with KEGG release 70 (Kanehisa et al. 2012), using the default cutoff E value of 10^-5^. For pathway comparison, predicted proteomes of the assembly and other trematodes were analyzed with GhostKoala (Kanehisa et al. 2016). Reconstructed pathways were recovered and compared with an inhouse script. Module completion was assessed as described (Tyagi et al. 2015b). Putative proteases and protease inhibitors were identified and classified using the online MEROPS peptidase database server (release 9.11) (Rawlings et al. 2014). For *F. hepatica,* transmembrane domains and classical secretion peptides were predicted using Phobius (Kall et al. 2004; Kall et al. 2007). For *nFh*, secretion signals and transmembrane domains were predicted using SignalP 4.1 (Petersen et al. 2011). Candidates for secreted proteins into the extracellular space were considered if signal peptide and no transmembrane domains were predicted. Complete functional annotation of the inferred proteins of *F. hepatica* and *nFh* are provided in Table S4 and Table S6, respectively.

## MultiParanoid analyses of *F. hepatica* and *nFh* gene families

Inferred protein sequences of *F hepatica* were compared to proteins from other trematodes (from WormBase ParaSite: *F. hepatica* UK, [PRJEB6687](http://www.ncbi.nlm.nih.gov/bioproject/PRJEB6687); *C. sinensis*, PRJDA72781; *O. viverrini,* PRJNA222628; *Schistosoma haematobium*, PRJNA78265; *Schistosoma japonicum*, PRJEA34885; *Schistosoma mansoni*, PRJEA36577; *Schmidtea mediterranea,* PRJNA12585) and cognate mammalian hosts (from Ensembl: *Homo sapiens*, GRCh38; *Bos taurus*, UMD3.1; *Ovis aries*, Oar_v3.1) using InParanoid version 4.1 (Remm et al. 2001; O'Brien et al. 2005). In the same manner, inferred protein sequences from *Neorickettsia* were compared to proteins from representative species from the Anaphasmataceae, included all four fully sequenced *Neorickettsia* (from NCBI RefSeq: *N. helminthoeca*, NZ_CP007481.1; *N. risticii*, NC_013009.1; *N. sennetsu*, NC_007798.1; *Ehrlichia chaffeensis*, NC_007799.1; *Anaplasma phagocytophilum*, NC_007797.1; *Wolbachia* endosymbiont of *D. melanogaster*, NC_002978.6; *Wolbachia* endosymbiont of *Brugia malayi*, NC_006833.1). Orthologous protein families (OPFs) were constructed from pairwise InParanoid comparisons using MultiParanoid (Alexeyenko et al. 2006).

More detailed phylogenetic analysis was performed on selected OPFs. Briefly, gene models identified as members of each family were used as baits to collect the orthologous from the TreeFam tool available at WormBase ParaSite. The corresponding sequences were retrieved and aligned with MAFF (Katoh and Standley 2013), and maximum likelihood trees were generated with MEGA (Tamura et al. 2013), and visualized with Evolview (Zhang et al. 2012).

## *F. hepatica* RNAseq and expression analyses

Total RNA was extracted from eggs and adults from the gall bladder of naturally infected sheep and metacercariae (Baldwin Aquatics Inc., Monmouth, Oregon) using TRIzol reagent (Invitrogen/Life Technologies, Carlsbad, CA) according to the manufacturer’s instructions and treated with Ambion Turbo DNase (Ambion/Applied Biosystems, Austin, TX). RNA quality and yield were assessed using Agilent 2100 Bioanalyzer (Agilent Technologies, Cedar Creek, Texas), and NanoDrop ND-1000 (NanoDrop Technologies, Wilmington, DE), respectively. The purified RNA was poly(A) selected using the MicroPoly(A)Purist™ Kit (Ambion/Applied Biosystems, Austin, TX) and reverse transcribed using the Ovation® RNA-Seq V2 kit (NuGen Technologies, Inc., San Carlos, CA). Paired-end cDNA libraries were generated according to standard protocols and sequenced on the Illumina HiSeq 2000 platform. Adapter sequences and low complexity regions were trimmed, reads were filtered based on length, sequence complexity, and similarity to suspected contaminants (McNulty et al. 2014). Remaining, high-quality RNAseq reads were aligned to the genome assembly using Tophat2 (version 2.0.8, default parameters, (Kim et al. 2013)) using the genome annotation (gff3) as a guide. Genes were considered expressed if RNAseq reads covered at least half of the gene length (≥50% breadth of coverage), as determined by in house tool Refcov. The number of reads associated with each gene was determined by HTSeq-Count, and differential gene expression analyses were carried out using the DESeq2 package, version 1.6.1, (Anders and Huber 2010) as recommended according to the authors' established protocol (population-adjusted p-value cutoff of 0.1) (Anders et al. 2013). Genes were considered constitutively expressed when they (1) showed ≥50% breadth of coverage in all samples and (2) no evidence of differential expression.

## Phylogenetic analysis of *Neorickettsia*

The 16S rRNA sequence of *nFh* - identified during feature prediction and confirmed by BLAST comparison with the 16S rRNA sequences of other *Neorickettsia* species - was aligned to those of other clade III *Neorickettsia* (Greiman et al. 2014) using an implementation of ClustalW within MEGA6 (Tamura et al. 2013). Sequences were trimmed to remove unaligned portions since most available 16S rRNA gene sequences are incomplete. MrModeltest2.3 was used to determine the best-fit model prior to phylogenetic analysis according to the Akiake information criterion (Nyander 2004). MrBayes v3.2.1 (Ronquist et al. 2012) was executed using 4 MCMC chains and 1,000,000 generations with sampling every 1,000 generations. Bayesian posterior probabilities were determined after discarding the first 1,000 trees as burn-in.

A total of 473 conserved, single-copy gene OPFs were identified during the MultiParanoid analysis. The proteins from each of these OPFs were aligned using Muscle v3.7 (Edgar 2004), and the 473 individual protein alignments were concatenated using FASconCAT v1.0 (Kuck and Meusemann 2010). MrBayes v3.2.1 (Ronquist et al. 2012) was executed using 4 MCMC chains and 2,000 generations with sampling every 100 generations. Bayesian posterior probabilities were determined after discarding the first 20 trees as burn-in.

## Statistical enrichment analyses

GO term enrichment in gene sets relative to all genes in the species of interest (*F. hepatica* or its *Neorickettsia* endobacterium) was determined using FUNC (Prufer et al. 2007), which considers the hierarchical structure of Gene Ontology and corrects for multiple testing. Statistical enrichment of other gene sets (e.g. testing enrichment of conserved genes among differentially expressed genes) was performed using a non-parametric binomial distribution test, with FDR correction for multiple testing where appropriate.

## Histological examination of *F. hepatica* and its *Neorickettsia* endobacterium

Oregon strain flukes from sheep were fixed first in 70% ethanol and then in 10% buffered formalin overnight, tissue processed (Shandon 1000 Tissue Processor, Thermo Scientific, Waltham, MA, USA), embedded in paraffin, sectioned at 5 μm and routinely stained with Hematoxylin & Eosin. *Neorickettsia*-negative flukes from naturally infected sheep in Uruguay were processed identically. Unstained tissue sections were rehydrated and blocked in 5% rabbit serum (30 minutes) to prevent non-specific antibody binding. Polyclonal mouse antisera raised against a recombinant *Neorickettsia* surface protein from *Plagiorchis elegans* (Genbank Accession KX082665, *PeN*sp-3) diluted 1:250 in phosphate buffered saline containing 0.1% Triton-X and 1% bovine serum albumin was used as the primary antibody. Anti-mouse IgG Alexa Fluor 488 (Invitrogen) was used as a secondary antibody for fluorescence microscopy. Wheat germ agglutinin 633 (200 µg/ml, Invitrogen, Carlsbad, CA, USA) and DAPI (Prolong Antifade with DAPI, Molecular Probes by Life Technologies, Carlsbad, CA, USA) were used to label membranes and double-stranded DNA, respectively. Sections were examined using a wide field fluorescence microscope (WFFM, Zeiss Axios Imager Upright Fluorescence Microscope) with plan-apochromat 100X oil, 63X or 40X objectives. Fluorescence microscopy was performed at the Washington University Molecular Microbiology Imaging Facility (http://micro.imaging.wustl.edu/).

# Supplementary References

Alexeyenko A, Tamas I, Liu G, Sonnhammer EL. 2006. Automatic clustering of orthologs and inparalogs shared by multiple proteomes. *Bioinformatics* **22**: e9-15.

Anders S, Huber W. 2010. Differential expression analysis for sequence count data. *Genome Biol* **11**: R106.

Anders S, McCarthy DJ, Chen Y, Okoniewski M, Smyth GK, Huber W, Robinson MD. 2013. Count-based differential expression analysis of RNA sequencing data using R and Bioconductor. *Nat Protoc* **8**: 1765-1786.

Angiuoli SV, Gussman A, Klimke W, Cochrane G, Field D, Garrity G, Kodira CD, Kyrpides N, Madupu R, Markowitz V et al. 2008. Toward an online repository of Standard Operating Procedures (SOPs) for (meta)genomic annotation. *OMICS* **12**: 137-141.

Ashburner M, Ball CA, Blake JA, Botstein D, Butler H, Cherry JM, Davis AP, Dolinski K, Dwight SS, Eppig JT et al. 2000. Gene ontology: tool for the unification of biology. The Gene Ontology Consortium. *Nat Genet* **25**: 25-29.

Boetzer M, Henkel CV, Jansen HJ, Butler D, Pirovano W. 2011. Scaffolding pre-assembled contigs using SSPACE. *Bioinformatics* **27**: 578-579.

Boetzer M, Pirovano W. 2012. Toward almost closed genomes with GapFiller. *Genome Biol* **13**: R56.

Cancela M, Ruetalo N, Dell'Oca N, da Silva E, Smircich P, Rinaldi G, Roche L, Carmona C, Alvarez-Valin F, Zaha A et al. 2010. Survey of transcripts expressed by the invasive juvenile stage of the liver fluke Fasciola hepatica. *BMC Genomics* **11**: 227.

Cantarel BL, Korf I, Robb SM, Parra G, Ross E, Moore B, Holt C, Sanchez Alvarado A, Yandell M. 2008. MAKER: an easy-to-use annotation pipeline designed for emerging model organism genomes. *Genome Res* **18**: 188-196.

Cwiklinski K, Dalton JP, Dufresne PJ, La Course J, Williams DJ, Hodgkinson J, Paterson S. 2015. The Fasciola hepatica genome: gene duplication and polymorphism reveals adaptation to the host environment and the capacity for rapid evolution. *Genome Biol* **16**: 71.

Edgar RC. 2004. MUSCLE: multiple sequence alignment with high accuracy and high throughput. *Nucleic Acids Res* **32**: 1792-1797.

Friedländer MR, Mackowiak SD, Li N, Chen W, Rajewsky N. 2012. miRDeep2 accurately identifies known and hundreds of novel microRNA genes in seven animal clades. *Nucleic Acids Res* **40**: 37-52.

Gnerre S, Maccallum I, Przybylski D, Ribeiro FJ, Burton JN, Walker BJ, Sharpe T, Hall G, Shea TP, Sykes S et al. 2011. High-quality draft assemblies of mammalian genomes from massively parallel sequence data. *Proc Natl Acad Sci U S A* **108**: 1513-1518.

Greiman SE, Tkach VV, Pulis E, Fayton TJ, Curran SS. 2014. Large scale screening of digeneans for Neorickettsia endosymbionts using real-time PCR reveals new Neorickettsia genotypes, host associations and geographic records. *PLoS One* **9**: e98453.

Griffiths-Jones S, Bateman A, Marshall M, Khanna A, Eddy SR. 2003. Rfam: an RNA family database. *Nucleic Acids Res* **31**: 439-441.

Hunter S, Jones P, Mitchell A, Apweiler R, Attwood TK, Bateman A, Bernard T, Binns D, Bork P, Burge S et al. 2012. InterPro in 2011: new developments in the family and domain prediction database. *Nucleic Acids Res* **40**: D306-312.

Jurka J, Kapitonov VV, Pavlicek A, Klonowski P, Kohany O, Walichiewicz J. 2005. Repbase Update, a database of eukaryotic repetitive elements. *Cytogenet Genome Res* **110**: 462-467.

Kall L, Krogh A, Sonnhammer EL. 2004. A combined transmembrane topology and signal peptide prediction method. *J Mol Biol* **338**: 1027-1036.

Kall L, Krogh A, Sonnhammer EL. 2007. Advantages of combined transmembrane topology and signal peptide prediction--the Phobius web server. *Nucleic Acids Res* **35**: W429-432.

Kanehisa M, Goto S, Sato Y, Furumichi M, Tanabe M. 2012. KEGG for integration and interpretation of large-scale molecular data sets. *Nucleic Acids Res* **40**: D109-114.

Kanehisa M, Sato Y, Morishima K. 2016. BlastKOALA and GhostKOALA: KEGG Tools for Functional Characterization of Genome and Metagenome Sequences. *J Mol Biol* **428**: 726-731.

Katoh K, Standley DM. 2013. MAFFT multiple sequence alignment software version 7: improvements in performance and usability. *Mol Biol Evol* **30**: 772-780.

Kim D, Pertea G, Trapnell C, Pimentel H, Kelley R, Salzberg SL. 2013. TopHat2: accurate alignment of transcriptomes in the presence of insertions, deletions and gene fusions. *Genome Biol* **14**: R36.

Kohany O, Gentles AJ, Hankus L, Jurka J. 2006. Annotation, submission and screening of repetitive elements in Repbase: RepbaseSubmitter and Censor. *BMC Bioinformatics* **7**: 474.

Korf I. 2004. Gene finding in novel genomes. *BMC Bioinformatics* **5**: 59.

Kozomara A, Griffiths-Jones S. 2014. miRBase: annotating high confidence microRNAs using deep sequencing data. *Nucleic Acids Res* **42**: D68-73.

Krzywinski MI, Schein JE, Birol I, Connors J, Gascoyne R, Horsman D, Jones SJ, Marra MA. 2009. Circos: An information aesthetic for comparative genomics. *Genome Res* doi:10.1101/gr.092759.109.

Kuck P, Meusemann K. 2010. FASconCAT: Convenient handling of data matrices. *Mol Phylogenet Evol* **56**: 1115-1118.

Lagesen K, Hallin P, Rodland EA, Staerfeldt HH, Rognes T, Ussery DW. 2007. RNAmmer: consistent and rapid annotation of ribosomal RNA genes. *Nucleic Acids Res* **35**: 3100-3108.

Li H, Durbin R. 2009. Fast and accurate short read alignment with Burrows-Wheeler transform. *Bioinformatics* **25**: 1754-1760.

Lowe TM, Eddy SR. 1997. tRNAscan-SE: a program for improved detection of transfer RNA genes in genomic sequence. *Nucleic Acids Res* **25**: 955-964.

McNulty SN, Fischer PU, Townsend RR, Curtis KC, Weil GJ, Mitreva M. 2014. Systems biology studies of adult paragonimus lung flukes facilitate the identification of immunodominant parasite antigens. *PLoS Negl Trop Dis* **8**: e3242.

McNulty SN, Strube C, Rosa BA, Martin JC, Tyagi R, Choi YJ, Wang Q, Hallsworth Pepin K, Zhang X, Ozersky P et al. 2016. Dictyocaulus viviparus genome, variome and transcriptome elucidate lungworm biology and support future intervention. *Sci Rep* **6**: 20316.

Nyander JAA. 2004. MrModeltest v2. In *Program distributed by the author*. Evolutionary Biology Centre, Uppsala University, Sweden.

O'Brien KP, Remm M, Sonnhammer EL. 2005. Inparanoid: a comprehensive database of eukaryotic orthologs. *Nucleic Acids Res* **33**: D476-480.

O'Leary NA, Wright MW, Brister JR, Ciufo S, Haddad D, McVeigh R, Rajput B, Robbertse B, Smith-White B, Ako-Adjei D et al. 2016. Reference sequence (RefSeq) database at NCBI: current status, taxonomic expansion, and functional annotation. *Nucleic Acids Res* **44**: D733-745.

Petersen TN, Brunak S, von Heijne G, Nielsen H. 2011. SignalP 4.0: discriminating signal peptides from transmembrane regions. *Nat Methods* **8**: 785-786.

Prufer K, Muetzel B, Do H, Weiss G, Khaitovich P, Rahm E, Paabo S, Lachmann M, Enard W. 2007. FUNC: a package for detecting significant associations between gene sets and ontological annotations. *BMC Bioinformatics* **8**: 41.

Quevillon E, Silventoinen V, Pillai S, Harte N, Mulder N, Apweiler R, Lopez R. 2005. InterProScan: protein domains identifier. *Nucleic Acids Res* **33**: W116-120.

Rawlings ND, Waller M, Barrett AJ, Bateman A. 2014. MEROPS: the database of proteolytic enzymes, their substrates and inhibitors. *Nucleic Acids Res* **42**: 23.

Remm M, Storm CE, Sonnhammer EL. 2001. Automatic clustering of orthologs and in-paralogs from pairwise species comparisons. *J Mol Biol* **314**: 1041-1052.

Robinson MW, Menon R, Donnelly SM, Dalton JP, Ranganathan S. 2009. An integrated transcriptomics and proteomics analysis of the secretome of the helminth pathogen Fasciola hepatica: proteins associated with invasion and infection of the mammalian host. *Mol Cell Proteomics* **8**: 1891-1907.

Ronquist F, Teslenko M, van der Mark P, Ayres DL, Darling A, Hohna S, Larget B, Liu L, Suchard MA, Huelsenbeck JP. 2012. MrBayes 3.2: efficient Bayesian phylogenetic inference and model choice across a large model space. *Syst Biol* **61**: 539-542.

Salamov AA, Solovyev VV. 2000. Ab initio gene finding in Drosophila genomic DNA. *Genome Res* **10**: 516-522.

Stanke M, Diekhans M, Baertsch R, Haussler D. 2008. Using native and syntenically mapped cDNA alignments to improve de novo gene finding. *Bioinformatics* **24**: 637-644.

Tamura K, Stecher G, Peterson D, Filipski A, Kumar S. 2013. MEGA6: Molecular Evolutionary Genetics Analysis version 6.0. *Mol Biol Evol* **30**: 2725-2729.

Tang YT, Gao X, Rosa BA, Abubucker S, Hallsworth-Pepin K, Martin J, Tyagi R, Heizer E, Zhang X, Bhonagiri-Palsikar V et al. 2014. Genome of the human hookworm Necator americanus. *Nat Genet* doi:10.1038/ng.2875.

Tyagi R, Joachim A, Ruttkowski B, Rosa BA, Martin JC, Hallsworth-Pepin K, Zhang X, Ozersky P, Wilson RK, Ranganathan S et al. 2015a. Cracking the nodule worm code advances knowledge of parasite biology and biotechnology to tackle major diseases of livestock. *Biotechnology advances* **33**: 980-991.

Tyagi R, Rosa BA, Lewis WG, Mitreva M. 2015b. Pan-phylum Comparison of Nematode Metabolic Potential. *PLoS Negl Trop Dis* **9**: e0003788.

Wilson RA, Wright JM, de Castro-Borges W, Parker-Manuel SJ, Dowle AA, Ashton PD, Young ND, Gasser RB, Spithill TW. 2011. Exploring the Fasciola hepatica tegument proteome. *Int J Parasitol* **41**: 1347-1359.

Young ND, Hall RS, Jex AR, Cantacessi C, Gasser RB. 2010. Elucidating the transcriptome of Fasciola hepatica - a key to fundamental and biotechnological discoveries for a neglected parasite. *Biotechnol Adv* **28**: 222-231.

Zerbino DR, Birney E. 2008. Velvet: algorithms for de novo short read assembly using de Bruijn graphs. *Genome Res* **18**: 821-829.

Zhang H, Gao S, Lercher MJ, Hu S, Chen WH. 2012. EvolView, an online tool for visualizing, annotating and managing phylogenetic trees. *Nucleic Acids Res* **40**: W569-572.
